# A novel feedback loop between DYRK2 and USP28 regulates cancer homeostasis and DNA damage signaling

**DOI:** 10.1038/s41418-025-01565-w

**Published:** 2025-08-26

**Authors:** Lucía Suanes-Cobos, Irene Aguilera-Ventura, Miguel Torres-Ramos, Alejandra Serrano-Yubero, Claudia Moreno Fernández-Aliseda, Silvia Fernández, Martín Garrido-Rodríguez, Susana de la Luna, Cristian Prieto-Garcia, Markus E. Diefenbacher, Ernesto Mejías-Pérez, Marco A. Calzado

**Affiliations:** 1 Instituto Maimónides de Investigación Biomédica de Córdoba (IMIBIC), Córdoba, Spain; 2 Departamento de Biología Celular, Fisiología e Inmunología, Universidad de Córdoba, Córdoba, Spain; 3 Hospital Universitario Reina Sofía, Córdoba, Spain; 4 Genome Biology Unit, European Molecular Biology Laboratory, Heidelberg, Germany; 5 Institute for Computational Biomedicine, Heidelberg University and Heidelberg University Hospital, Heidelberg, Germany; 6 Centre for Genomic Regulation (CRG), The Barcelona Institute of Science and Technology (BIST), Barcelona, Spain; 7 Centro de Investigación Biomédica en Red en Enfermedades Raras (CIBERER), Barcelona, Spain; 8 Institució Catalana de Recerca i Estudis Avançats (ICREA), Barcelona, Spain; 9 Protein Stability and Cancer Group, Department of Biochemistry and Molecular Biology, Theodor-Boveri-Institute, Biocenter, Würzburg, Germany; 10 Institute of Biochemistry II, Goethe University Frankfurt, Frankfurt am Main, Germany; 11 Institute of Lung Health and Immunity, Helmholtz Center Munich, Germany and German Center for Lung Research, DZL, Munich, Germany; 12 Ludwig Maximilian University Munich, Munich, Germany; 13 DKTK Munich, Munich, Germany

**Keywords:** Cell biology, Biochemistry, Kinases, Oncogenes

## Abstract

Posttranslational modifications, such as ubiquitination and phosphorylation, play pivotal roles in regulating protein stability in response to cellular stress. Dual-specificity tyrosine phosphorylation-regulated kinase 2 (DYRK2) and ubiquitin-specific peptidase 28 (USP28) are critical regulators of cell cycle progression, DNA damage response, and oncogenic signaling. However, their functional interplay remains largely unexplored. Here, we describe a novel bidirectional regulatory mechanism between DYRK2 and USP28 that integrates DNA damage response and ubiquitin-mediated protein degradation. We demonstrate that DYRK2 phosphorylates USP28, promoting its ubiquitination and proteasomal degradation in a kinase activity-independent manner, thereby contributing to the maintenance of oncogenic protein homeostasis. Conversely, USP28 functions as a deubiquitinase for DYRK2, stabilizing its protein levels and enhancing its kinase activity. Notably, we show that DYRK2 interacts and co-localizes with USP28, with the 521–541 DYRK2 region, particularly residue T525, playing a crucial role in USP28-mediated DYRK2 stabilization. Functionally, this reciprocal regulation modulates p53 signaling, influencing apoptotic responses to DNA damage. DYRK2-mediated phosphorylation of p53 at S46 is significantly reduced upon USP28 depletion, suggesting that USP28 facilitates DYRK2-dependent apoptosis. Additionally, our results highlight a complex regulatory axis involving USP28 and DYRK2, with implications for oncogenic cell death and genomic stability. Overall, our findings uncover a novel feedback loop in which DYRK2 and USP28 dynamically regulate each other to control proto-oncoprotein homeostasis and DNA damage signaling. This interplay offers potential therapeutic opportunities for targeting cancers with dysregulated ubiquitination and genomic instability.

## Introduction

Ubiquitination is a key posttranslational modification regulating protein degradation, cell cycle, DNA repair, and transcription [1]. This process is finely tuned by the dynamic interplay between ubiquitin ligases and deubiquitinating enzymes (DUBs). Among DUBs, the ubiquitin-specific protease (USP) family stands out due to its size and functional diversity [2, 3]. Ubiquitin-specific peptidase 28 (USP28), a member of this family, plays a crucial role in protein stability, cellular proliferation, DNA damage response, and oncogenesis [4].

USP28 preferentially cleaves K11-, K48-, and K63-linked ubiquitin chains, highlighting its specialized function [5]. Despite structural homology with USP25 [6, 7], USP28 is nuclear-localized and stabilizes key proteins in cancer, including c-Myc, p53, and CHK2 [8, 9]. Its regulation involves posttranslational modifications, such as phosphorylation and sumoylation, as well as interactions with regulatory proteins and microRNAs [4]. Phosphorylation by ATM enhances USP28 activity in response to DNA damage, whereas sumoylation inhibits it [10, 11]. Additionally, oncogenic factors like c-Jun and c-Myc regulate USP28 transcription, linking it to proliferation and transformation [12]. A defining feature of USP28 is its antagonistic relationship with the E3 ubiquitin ligase FBXW7, stabilizing oncoproteins such as c-Myc and NOTCH1, a mechanism particularly relevant in cancers characterized by FBXW7 depletion [13, 14]. Moreover, USP28 is essential for the DNA damage response (DDR), stabilizing 53BP1, MDC1, and CLASPIN to ensure checkpoint activation and repair [10, 15]. These roles underscore USP28 as a potential therapeutic target, with regulation models suggesting its dependence on E3 ligases for substrate recognition or phosphorylation-based activity control [4].

The Dual-specificity tyrosine phosphorylation-regulated kinase 2 (DYRK2), a member of the DYRK family within the CMGC kinase group, is distinct in its dual function as a serine/threonine kinase and a scaffold for the EDVP E3 ubiquitin ligase complex [16, 17]. DYRK2 plays a pivotal role in cell division, tissue development, and embryogenesis [18]. DYRK2 acts as both a tumor suppressor and a potential oncogene, phosphorylating key regulators such as p53, c-Jun, c-Myc, HSF1, CDC25A, and NOTCH1 [19–23]. DYRK2 also regulates FBXW7 stability and promotes the degradation of oncoproteins such as c-Jun, c-Myc, mTOR, and NOTCH1 [24]. Interestingly, USP28 shares functional similarities with DYRK2, having both dual regulatory loops with FBXW7 and common substrates [17, 25], yet their potential relationship remains unexplored.

Here, we describe a novel dual regulatory mechanism between DYRK2 and USP28, two key modulators of cellular stress responses and homeostasis. DYRK2 phosphorylates USP28, promoting its ubiquitination and proteasomal degradation. Conversely, USP28 deubiquitinates DYRK2 in response to DNA damage, controlling its stability and kinase activity leading to p53 pro-apoptotic phosphorylation. This reciprocal interplay forms a feedback loop that integrates DNA damage response and stress signaling, underscoring its role in maintaining cellular homeostasis under genotoxic stress.

## Materials and methods

### Cell culture, transfection, and reagents

HEK-293T, HeLa, CHO, A549, MDA-MB-468 (WT/DYRK2^–/–^) cells were cultured in Dulbecco’s Modified Eagle’s medium (DMEM). HCT116 (WT/FBXW7^–/–^) cells were cultured in McCoy’s 5A medium. SK-MES-1 and A427 cells were cultured in Eagle’s Minimum Essential Medium (EMEM). H460 cells were cultured in Roswell Park Memorial Institute (RPMI) 1640 Medium. See Supplementary Methods for the BEAS-2B squamous-cell differentiation model. All culture media were supplemented with 10% fetal bovine serum, (FBS), and 1% (v/v) penicillin/streptomycin (Sigma-Aldrich, St Louis, Missouri, USA). Cells were grown at 37 °C and 5% CO_2_, and were routinely tested for Mycoplasma and its authentication were determined using GenePrint10 System (Promega, Madison, Wisconsin, USA).

Transient transfection was performed using polyethylenimine, except for siRNAs experiments for which Lipofectamine 2000 (Invitrogen, Waltham, Massachusetts, USA) was used. To perform site-directed mutagenesis QuikChange II Site-Directed Mutagenesis Kit (Agilent Technologies, California, USA) was used with specific designed primers described in Supplementary Table 1. Reagents and plasmids used in this work are listed in Supplementary Tables 2 and 3, respectively.

### Western blotting (WB)

Cells were washed twice with phosphate buffered saline (PBS) and soluble extracts were prepared using NP-40 lysis buffer, at 36 h post transfection. For sodium dodecyl sulfate polyacrylamide gel electrophoresis (SDS-PAGE), the lysates were mixed with Laemmli buffer and boiled at 95 °C for 5 min. Then, proteins were transferred to nitrocellulose membranes using a semi-dry transfer. After blocking with non-fat milk 5% (w/v) in Tris Buffer Saline-0.1% Tween20 (TBS-T), membranes were incubated overnight at 4 °C with primary antibody diluted in 5% (w/v) milk in TBS-T. Incubation with secondary antibody was done for 1 h at room temperature. Protein detection was performed by fluorescence in ChemiDoc MP Imaging System machine (Bio-rad, California, USA). Antibodies and buffers used in this work are listed in Supplementary Tables 2 and 4.

### Immunoprecipitation

Cells were washed in PBS and lysed in IP buffer. Cell extracts were incubated with 2 μg of antibody overnight at 4 °C. Then, 50 μL protein A/G Sepharose (Santa Cruz, California, USA) was added for antibody isolation for 1 h at room temperature. Immunoprecipitates were washed five times in IP buffer. Then, proteins of interest were detected by WB. Buffer composition is described in Supplementary Table 4.

### Data analysis

Fluorescent WB band intensities were obtained with ImageJ v1.45 software. Results in bar graphs are represented as mean ± SD with individual values shown with dots. GraphPad Prism version 8.00 (GraphPad, San Diego, CA, USA) was used to determine statistical differences by 2-tailed unpaired Student’s *t*-test, being *P* < 0.05 considered as significant. Details for the generation of the 3D USP28 structure model and other methods employed in the article are provided in Supplementary Methods.

## Results

### DYRK2 regulates USP28 protein levels

**Fig. 1:**
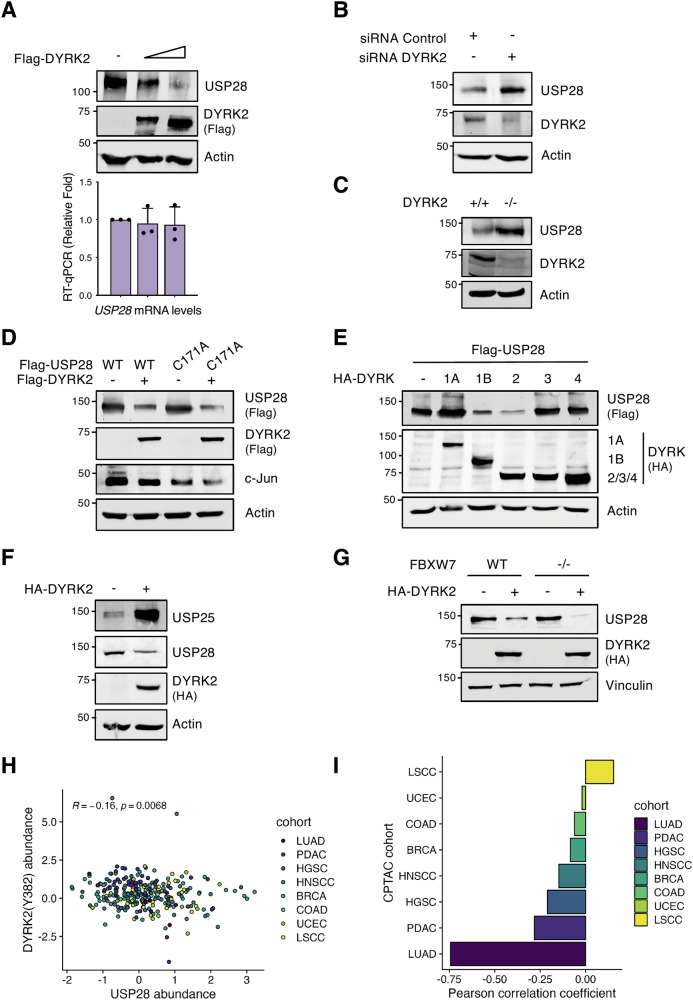
DYRK2 regulates USP28 protein levels. **A** HEK-293T cells were transfected with a gradient of Flag-DYRK2. Samples were split for WB (upper panel) or RT-qPCR (lower panel; mean ± SD, *n* = 3). **B** HEK-293T cells were transfected with siRNA-control or siRNA-DYRK2 and the protein level of USP28 and DYRK2 analyzed by WB. **C** The protein level of the indicated proteins was analyzed in MDA-MB-468 WT or DYRK2 knockout cells by WB. **D** HeLa cells were transfected with Flag-USP28 WT or inactive mutant (C171A) in presence or absence of Flag-DYRK2 and protein levels were analyzed by WB. **E**, **F** HEK-293T cells were transfected with the indicated plasmid and protein levels were determined by WB. **G** HCT116 WT or FBXW7 knockout cells were transfected or not with HA-DYRK2 and protein levels were analyzed by WB. **H** Correlation between active DYRK2 (Y382 phosphorylation state) and USP28 protein abundance across CPTAC pan-cancer data. Each point represents a patient, colored by the cancer cohort. The Pearson correlation coefficient and corresponding *P*-value are displayed in the top left corner. **I** Bar plot showing the Pearson correlation coefficient between active DYRK2 (Y382 phosphorylation state) and USP28 protein abundance across individual cancer cohorts. A significant negative correlation was observed only in the lung adenocarcinoma cohort (*P*-value = 7e–5). Note: a representative blot is shown of at least 3 biological replicates.

In a previous study, we described FBXW7 regulation by DYRK2 [24]. Given the converging roles of DYRK2 and USP28 in modulating FBXW7 and its substrates, we aimed to investigate a potential functional crosstalk between these two proteins. To this end, we first analyzed the mRNA and protein levels of USP28 in response to increasing DYRK2 expression. As shown in Fig. 1A, USP28 protein levels decreased in a DYRK2 dose-dependent manner, while mRNA levels remained unchanged. A similar effect was observed upon ectopic expression of both proteins (Fig. S1A). In contrast, DYRK2 depletion by specific siRNA (Fig. 1B) or CRISPR/Cas9 genetic deletion (Fig. 1C), increased USP28 protein levels. This effect was independent of USP28 catalytic activity, as DYRK2 also reduced levels of the catalytically inactive USP28 mutant (USP28^C171A^), impacting USP28 substrates like c-Jun (Fig. 1D).

Considering DYRK family conservation, we also examined other members. As shown in Fig. 1E, DYRK1B promoted USP28 downregulation, whereas DYRK1A markedly increased its stabilization. Moreover, given the high homology between USP28 and USP25 [6], we investigated whether DYRK2 similarly affects USP25. Unlike USP28, USP25 protein levels were stabilized by DYRK2 expression (Fig. 1F), indicating a divergent regulatory mechanism. Since FBXW7 is linked to USP28 and regulated by DYRK2 [24, 26], we examined its role in this mechanism. As shown in Fig. 1G, DYRK2-mediated USP28 downregulation persisted in HCT116 FBXW7-KO cells and was similarly observed upon ectopic co-expression of USP28 (Fig. S1B). Similarly, SCF^FBXW7^ complex inactivation using a dominant-negative CUL1 plasmid (dn-CUL1) produced comparable results (Fig. S1C), indicating that this process is FBXW7-independent. Finally, to investigate the relationship between active DYRK2 -using Y382 phosphorylation at the activation loop as marker- and USP28 abundance in cancer, we analyzed CPTAC phospho-proteomic and proteomic data [27]. A pan-cancer analysis revealed a trend toward a negative correlation between USP28 levels and the active form of DYRK2 across different tumor types (Fig. 1H), with high statistical significance primarily driven by lung adenocarcinoma (LUAD) (Fig. 1I). In summary, these findings indicate that DYRK2 reduces USP28 stability at the protein level, with a potential role in cancer.

### DYRK2 phosphorylates USP28 promoting its degradation through a kinase activity-independent mechanism

**Fig. 2:**
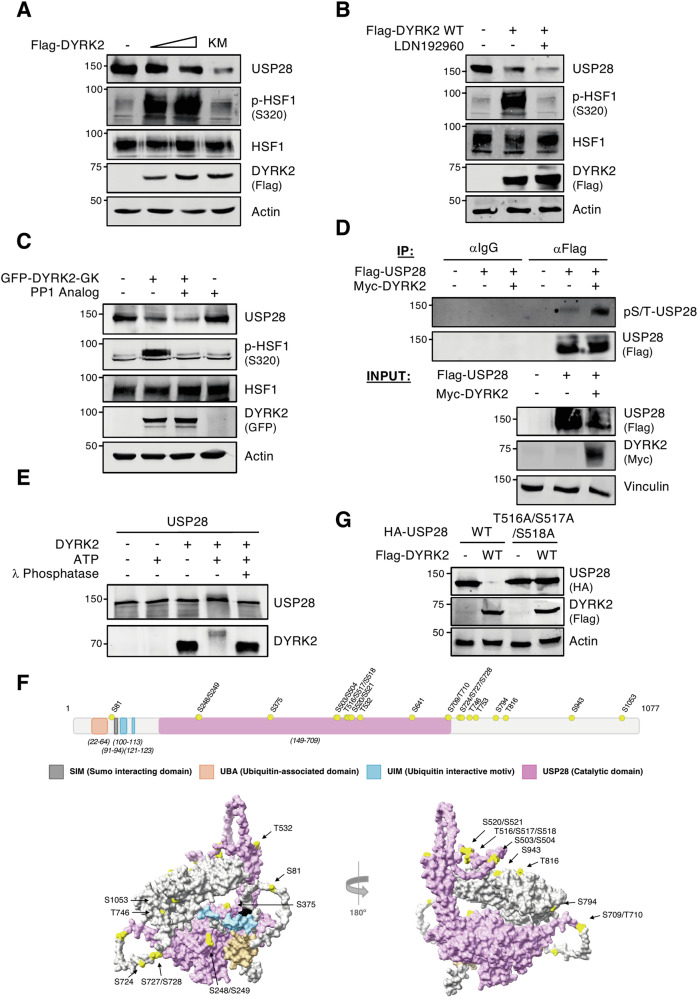
DYRK2 phosphorylates USP28 but promotes its degradation through a kinase activity-independent mechanism. **A** HEK-293T cells were transfected with a gradient of wild-type (WT) or kinase-dead version (KM) of Flag-DYRK2 and USP28 protein levels were determined by WB. **B** HeLa cells were transfected or not with Flag-DYRK2 WT and treated or not with the DYRK2 inhibitor LDN192960 (5 μM) for 2 h. **C** HeLa cells were transfected or not with a gatekeeper version of DYRK2 (GK) and treated or not with PP1 Analog (3 μM) for 3 h. In **A**, **B** and **C**, protein levels were analyzed by WB, and HSF1 phosphorylation at S320 was used as a control for DYRK2 activity. **D** MDA-MB-468 DYRK2-knockout cells were transfected with the indicated plasmids and treated with MG-132 (10 μM) for 12 h. USP28 Ser/Thr phosphorylation levels were analyzed by WB on the Flag-USP28 immunoprecipitates. **E** USP28 human recombinant protein was incubated in presence or absence of DYRK2 human recombinant protein, ATP and λ-phosphatase. Electrophoretic mobility was determined by WB. Changes in DYRK2 mobility are due to auto-phosphorylation. **F** 2D and 3D scheme of USP28 protein indicating the different motifs and domains of the protein in color code (see legend) and in yellow the phospho-residues identified by MS/MS in the presence of DYRK2. **G** HEK-293T cells were transfected with the indicated plasmids and analyzed by WB. Note: a significant result is shown of at least 3 biological replicates.

To determine whether DYRK2 enzymatic activity regulates USP28 protein levels, we analyzed its effects using the wild-type enzyme and a kinase-dead DYRK2 mutant (DYRK2 KM) on USP28 endogenous and ectopic levels. As shown in Figs. 2A and S2A, both DYRK2 versions produced comparable results. Similarly, DYRK2 chemical inhibition (Fig. 2B) and an analogue-sensitive DYRK2 mutant (DYRK2-GK), selectively inhibited by PP1 via a gatekeeper residue mutation in the ATP-binding pocket [20], yielded consistent findings (Figs. 2C and S2B). HSF1 phosphorylation served as a readout for DYRK2 kinase activity. Collectively, these results indicate that DYRK2 reduces USP28 levels independently of its kinase activity.

Since DYRK2 can regulate the accumulation of one of its substrates through both its kinase and scaffolding function [17], we could not rule out USP28 phosphorylation by DYRK2. To test this, we analyzed USP28 phosphorylation levels with and without DYRK2. As shown in Fig. 2D, USP28 phosphorylation increased in the presence of DYRK2. We then performed in vitro kinase assays with purified recombinant proteins. As shown in Fig. 2E, USP28 incubation with DYRK2 and ATP reduced its electrophoretic mobility, an effect reversed by λ-phosphatase, confirming direct phosphorylation by DYRK2.

To identify USP28 phosphorylation sites targeted by DYRK2, we performed mass spectrometry site identification (Fig. S2C). This analysis revealed 24 phosphorylated residues across the USP28 sequence (Fig. 2F). Several of these sites had been previously reported in low- and high-throughput in vivo studies [15, 28] and are documented in PhosphoSite (https://www.phosphosite.org). Most sites were solvent-exposed, except S641, T746, T753, and S1053, which reside behind flexible surface loops, potentially allowing exposure (Figs. 2F and S2D).

To identify residues involved in USP28 regulation by DYRK2, we selected 14 candidates based on MS/MS spectrum quality, phosphorylation frequency, USP25 vs USP28 regulation differences, functional annotations, and evolutionary conservation (Fig. S2E). We generated five non-phosphorylatable single-site mutants (S81A, S375A, T753A, T816A, S943A), three double mutants (S248A/S249A, S503A/S504A, S520A/S521A), and one triple mutant (T516A/S517A/S518A). All, except USP28-T516A/S517A/S518A, exhibited the same degradation pattern in response to DYRK2 (Figs. 2G and S2F). Notably, USP28-T516A/S517A/S518A still interacted with DYRK2, ruling out degradation loss due to structural disruption or impaired binding. In this sense, the USP28 triple mutant exhibits diminished binding to wild-type DYRK2, while its phosphomimetic counterpart (USP28-T516D/S517D/S518D) shows enhanced interaction (Fig. S2G). Although the USP28-T516A/S517A/S518A triple mutant is still phosphorylated in response to DYRK2 (Fig. S2H), it displays reduced total phosphorylation levels compared to the wild-type version (Fig. S2I), as well as a partial decrease in electrophoretic mobility in the presence of DYRK2 (Fig. S2J). All these data suggest a phosphorylation-dependent mechanism in which USP28 phosphorylation at T516/S517/S518 enhance DYRK2 wild-type specific substrate affinity.

The analysis of single mutants T516A, S517A or S518A failed to identify any one of them as the sole responsible residue, likely due to their proximity (Fig. S2K). In summary, DYRK2 phosphorylates USP28 at several residues increasing its interaction affinity but promotes its degradation via a kinase activity-independent mechanism.

### DYRK2 regulates USP28 via the ubiquitin-proteasome system

**Fig. 3:**
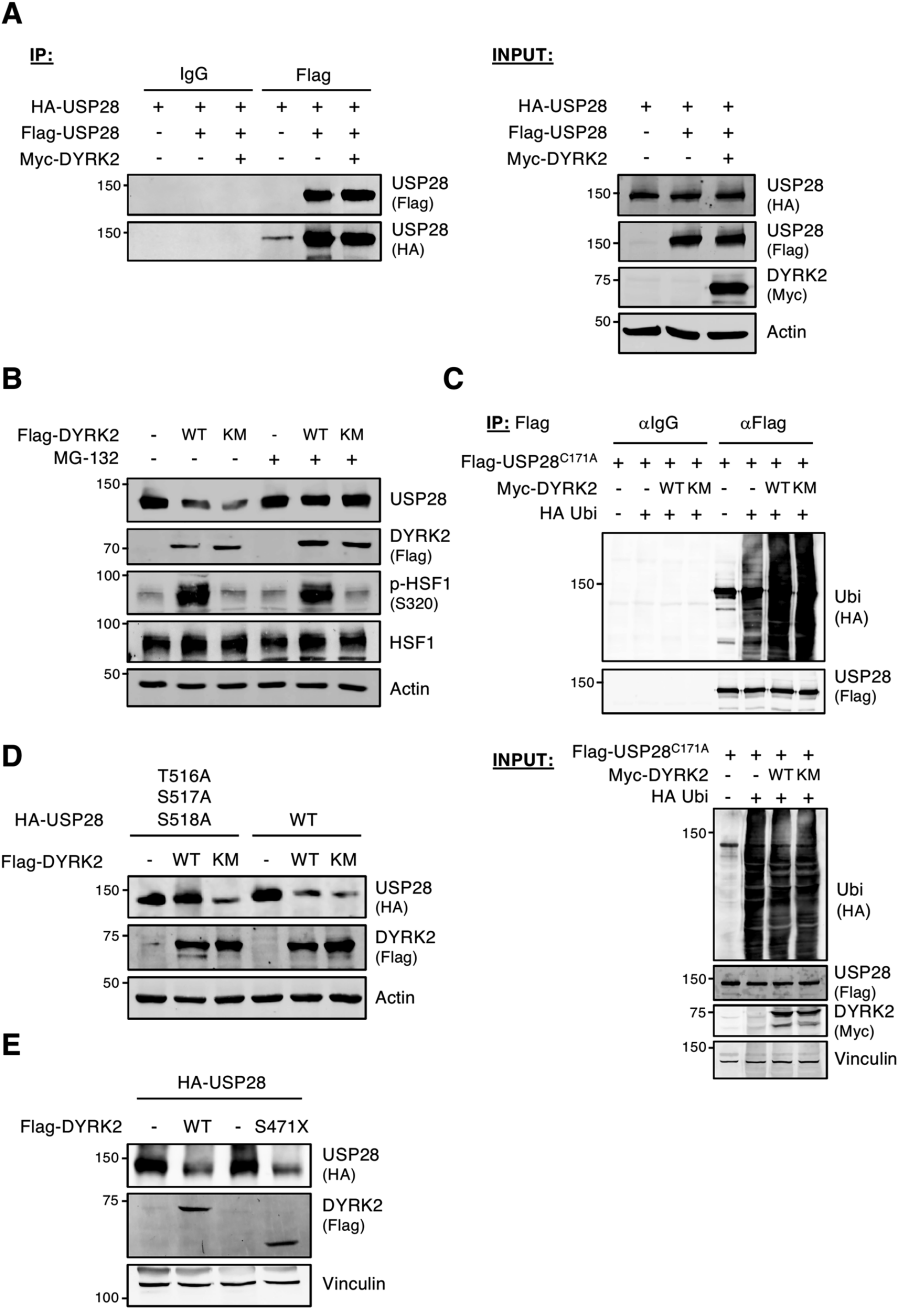
DYRK2 regulates USP28 stability via the ubiquitin-proteasome system. **A** Dimerization of USP28 assayed by co-immunoprecipitation of HEK-293T cells transfected with the indicated plasmids. The presence of the proteins in both in input lysates and the immunoprecipitates is shown by WB. **B** HeLa cells were transfected with the indicated plasmids and treated or not with MG-132 (10 μM) for 12 h. Protein levels were determined by WB. **C** HEK-293T cells were transfected with the indicated plasmids. Flag-USP28 inactive mutant (C171A) was immunoprecipitated and its ubiquitination profile was determined by WB. In **A** and **C**, extracts were prepared from cells treated with the proteasome inhibitor MG-132 (10 μM) for 12 h. **D**, **E** HEK-293T cells were transfected with the indicated plasmids and analyzed by WB. Note: all experiments were performed at least 3 times, and a representative blot is shown.

Since DYRK2-mediated substrate degradation via kinase-independent mechanisms is rare, we investigated this process in detail. First, we assessed USP28 half-life with and without DYRK2. As shown in Figs. S3A and S3B, DYRK2 expression reduced USP28 stability at both ectopic and endogenous levels. Since dimerization can influence USP28 activity on its substrate, we examined whether DYRK2 affects USP28 dimerization [29]. As shown in Fig. 3A, DYRK2 had no effect. To assess proteasome involvement, we analyzed USP28 levels in cells expressing wild-type (DYRK2 WT) or kinase-dead DYRK2 (DYRK2 KM) with or without the proteasome inhibitor MG-132. As shown in Figs. 3B and S3C, MG-132 blocked DYRK2-induced USP28 degradation. Consistently, DYRK2, regardless of its kinase activity, enhanced USP28 ubiquitination (Fig. 3C) and USP28-T516A/S517A/S518A mutant degradation (Fig. 3D). Finally, given DYRK2 scaffolding role in the EDVP complex [17, 25], we tested whether this function was required. Using a C-terminal truncated DYRK2 mutant (DYRK2-S471X), unable to EDVP scaffolding [17], we found that USP28 degradation was EDVP-independent (Fig. 3E). All these results demonstrate that DYRK2 regulates USP28 through a kinase activity-independent mechanism mediated by the proteasome.

### USP28 stabilizes DYRK2 protein levels

**Fig. 4:**
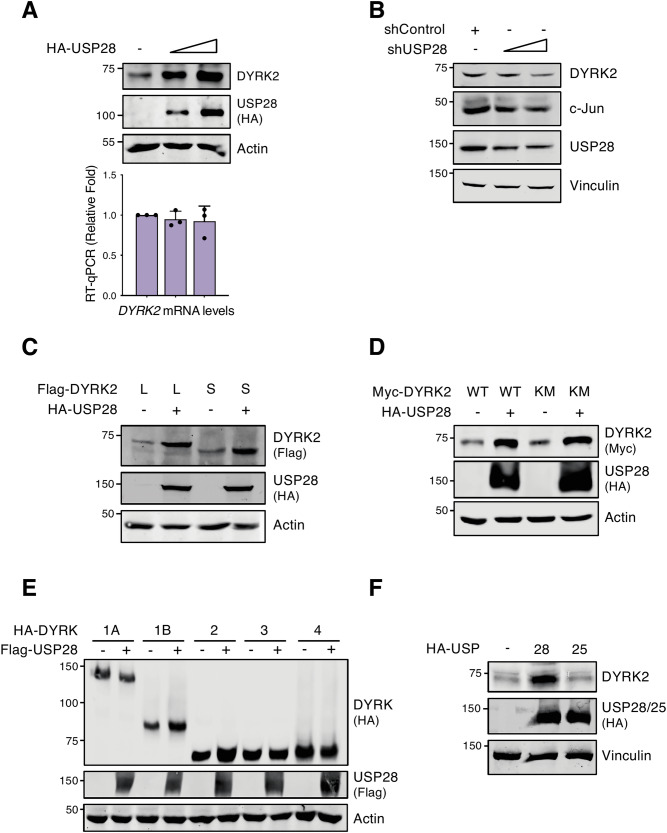
USP28 stabilizes DYRK2 protein levels. **A** HEK-293T cells were transfected with a gradient of HA-USP28. Samples were split for WB (upper panel) or RT-qPCR (lower panel) to determine DYRK2 protein or mRNA levels, respectively (mean ± SD, *n* = 3). **B** HEK-293T cells were transfected with the indicated shRNAs and the levels of the indicated proteins were determined by WB. c-Jun were used as control substrate. **C**–**F** HEK-293T cells were transfected with the indicated plasmids and protein levels were analyzed with specific antibodies by WB. In **C**, L and S indicate the 601 aa and 528 aa DYRK2 isoforms, respectively. Note: a representative blot was shown of at least 3 different biological replicates.

A reciprocal regulatory relationship between DYRK2 and several of its substrates has been reported [19, 30]. To assess whether this applies to USP28, we analyzed DYRK2 mRNA and protein levels in response to increasing USP28. As shown in Fig. 4A, DYRK2 protein levels increased dose-dependently, while mRNA levels remained unchanged. A similar effect was observed upon ectopic co-expression (Fig. S4A) and in different lung cancer cell lines (Fig. S4B). Conversely, USP28 knockdown via shRNA reduced DYRK2 protein levels (Fig. 4B) without affecting mRNA levels (Fig. S4C). DYRK2 is expressed as two splicing-derived proteoforms with distinct non-catalytic N-terminal domains [16]. The accumulation of the two proteoforms equally responded to USP28 (Fig. 4C). We also investigated whether DYRK2 kinase activity was required for the USP28-dependent stabilization. As shown in Fig. 4D, this effect was independent of DYRK2 kinase activity. Finally, to determine if this stabilizing effect extends to other DYRK members, we analyzed USP28 impact on additional DYRK proteins. Together with DYRK2, only DYRK1B showed stabilization (Fig. 4E). Additionally, it is important to highlight, DYRK2 stabilization was USP28-specific, as USP25 had no such similar effect (Figs. 4F and S4D).

### USP28 deubiquitinates DYRK2

**Fig. 5:**
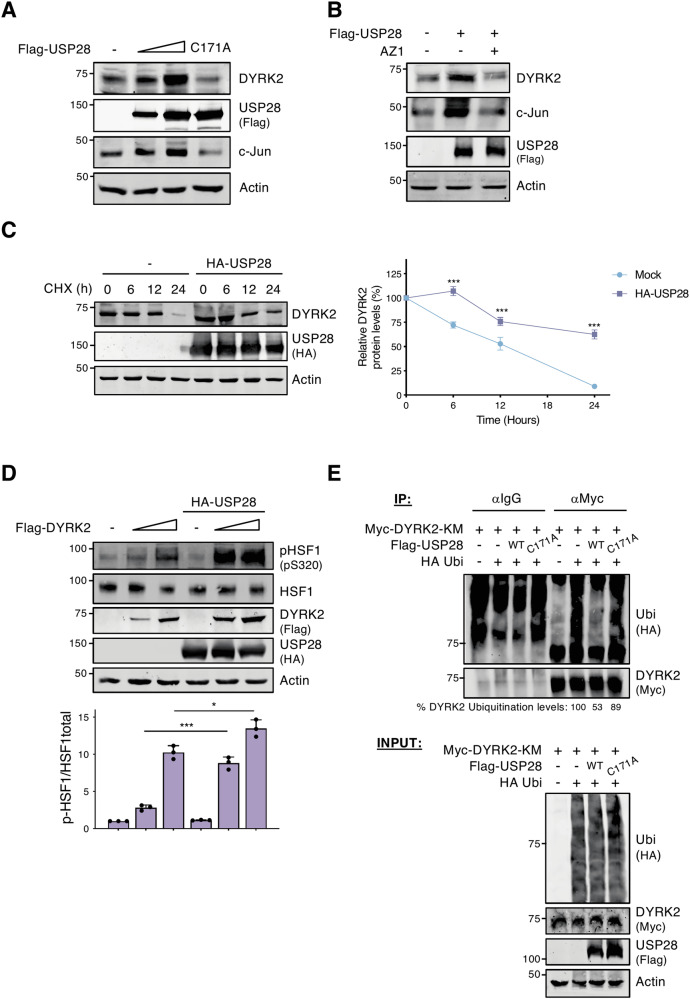
USP28 deubiquitinates DYRK2. **A** HEK-293T cells were transfected with a gradient of Flag-USP28 WT or inactive mutant (Flag-USP28^C171A^) and DYRK2 protein levels were analyzed by WB. **B** HEK-293T cells were transfected with the indicated plasmids and treated or not with the USP28 inhibitor AZ1 (10 μM) for 24 h. **C** HEK-29T cells were transfected or not with HA-USP28 and treated or not with Cycloheximide (CHX) (20 μg/mL) during the indicated times. Data represent mean ± SD of endogenous DYRK2 levels normalized with Actin, from 3 independent experiments (***P < 0.001). **D** DYRK2 activity was assessed with endogenous HSF1 phosphorylation (pS320) in HEK-293T cells in the presence/absence of HA-USP28. The bar plot represents mean ± SD of p-HSF1 band intensity normalized by HSF1 total band intensity from three independent experiments (***P < 0.001). **E** HEK-293T cells were transfected with the indicated plasmids and treated with MG-132 (10 μM) for 12 h. The ubiquitination profile of immunoprecipitated Myc-DYRK2 kinase mutant (KM) was determined by WB. The ubiquitination levels were quantified and presented as a percentage relative to the basal level in the absence of USP28 and normalized to the amount of immunoprecipitated DYRK2 (shown below the upper panel). In **A** and **B** c-Jun was used as a control of USP28 activity. Note: all experiments were performed at least 3 times, and a representative blot is shown.

To assess whether USP28 deubiquitinase activity stabilizes DYRK2, we analyzed DYRK2 levels with a catalytically inactive USP28 mutant (USP28^C171A^). Unlike wild-type USP28, this mutant not only failed to stabilize DYRK2 but also reduced its basal levels (Figs. 5A and S5A). To further confirm this effect, we treated cells with the USP28 inhibitor AZ1. As shown in Figs. 5B and S5B, AZ1 reversed USP28-mediated DYRK2 stabilization, confirming its enzymatic dependence. Finally, rescue experiments in USP28-inactive SK-MES-1 cells revealed that DYRK2 levels increased upon reintroduction of wild-type USP28 but remained unchanged with the catalytically active mutant (Fig. S5C).

Next, we examined whether USP28 regulates DYRK2 half-life. As shown in Figs. 5C and S5D, USP28 increased DYRK2 stability. This stabilization had functional consequences, as USP28 enhanced DYRK2 activity in a dose-dependent manner (Fig. 5D). Since USP28 could modulate DYRK2 posttranslationally via deubiquitination, we analyzed DYRK2 ubiquitination in the presence of wild-type and inactive USP28 (USP28^C171A^). As shown in Fig. 5E, wild-type USP28 reduced DYRK2 ubiquitination, an effect not observed with the inactive mutant. These findings demonstrate that USP28 deubiquitinates DYRK2, enhancing its stability and activity.

### DYRK2 interacts and co-localizes with USP28

**Fig. 6:**
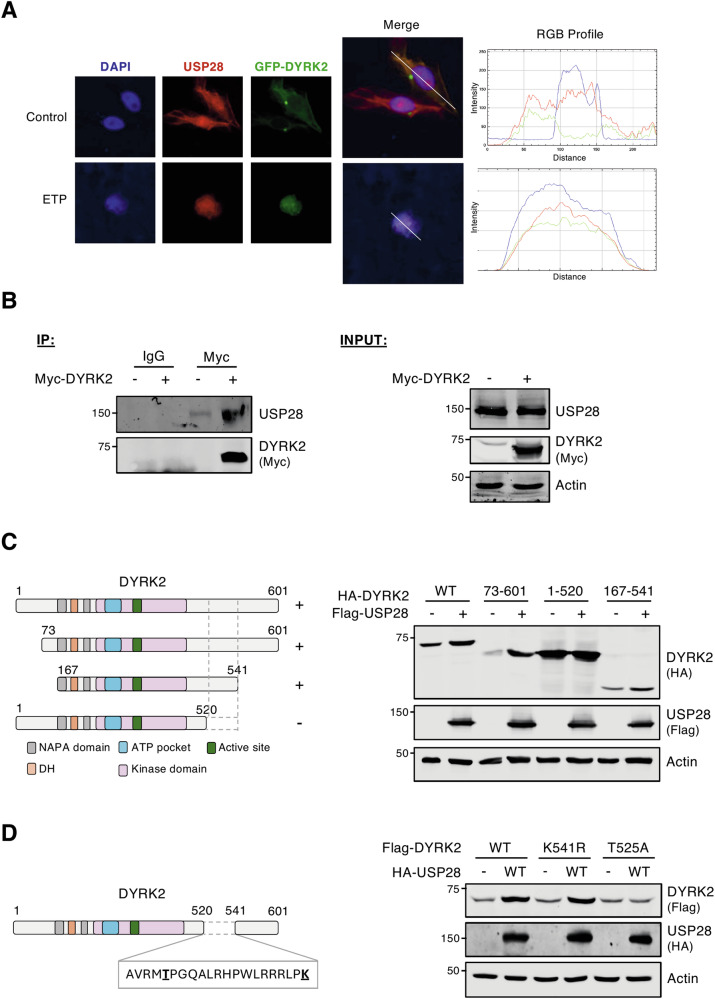
DYRK2 interacts and co-localizes with USP28. **A** CHO cells were transfected with a GFP-DYRK2 plasmid, fixed and analyzed by confocal microscopy using GFP-fluorescence or indirect immunofluorescence with a USP28 antibody after being treated with 10 μM MG-132 with or without 10 μM Etoposide (ETP) for 6 h. DAPI was used to stain DNA. Overlapping localization is shown in yellow (DYRK2/USP28) or purple (DYRK2/USP28/DNA). The RGB profiles indicate fluorescence intensity of each signal through the white line. **B** HEK-293T cells were transfected with the indicated plasmids, Myc-DYRK2 was immunoprecipitated and USP28 levels were determined by WB. Analysis of the USP28-dependent stabilization effects on DYRK2 in HEK-293T cells transfected with different versions of Flag-DYRK2 deletion mutants (**C**) or point-mutants (**D**) as shown in the schematic representations (left panels). Note: a representative blot is shown of at least 3 independent biological replicates.

Given the bidirectional regulatory relationship between DYRK2 and USP28, we next investigated whether their functional interaction depends on the formation of a stable complex. As shown in Fig. 6A, DYRK2 and USP28 predominantly co-localize in the cytoplasm under basal conditions. However, following DNA damage response, DYRK2 relocates to the nucleus and co-localizes with USP28 foci formed upon DNA damage (Fig. 6A). Furthermore, co-immunoprecipitation assays confirmed a robust interaction between DYRK2 and USP28 (Fig. 6B), being the interaction enhanced with the mutant version of DYRK2 without kinase activity (Fig. S6A).

To identify the DYRK2 region responsible for the USP28-dependent response, we performed domain-mapping experiments. As shown in Fig. 6C, a segment spanning amino acids 521–541 is essential for the USP28-mediated increased accumulation. To determine if this effect results from disrupted protein-protein interactions, we examined the binding capacity and subcellular localization of DYRK2 variants lacking this region. No significant alterations were observed in the DYRK2 deletion mutant compared to the wild-type protein in either the interaction assay (Fig. S6B) or subcellular localization (Fig. S6C), suggesting that this region does not mediate direct binding with USP28. We then sought to identify key residues for post-translational modifications within the 521–541 region. We selected threonine 525 and lysines 513 and 541 for further investigation and generated non-phosphorylatable (T525A) and non-ubiquitinatable (K513R and K541R) mutants (Figs. 6D and S6D). No changes were observed with the DYRK2 K513R and K541R mutants (Figs. 6D and S6D). In contrast, unlike wild-type DYRK2, the T525A mutant failed to stabilize in the presence of USP28 (Fig. 6D). These findings demonstrate that DYRK2 interacts and co-localizes with USP28, with the 521–541 region, particularly T525, being crucial for USP28-mediated DYRK2 stabilization.

### USP28 is required to DYRK2 stabilization in response to DNA damage

**Fig. 7:**
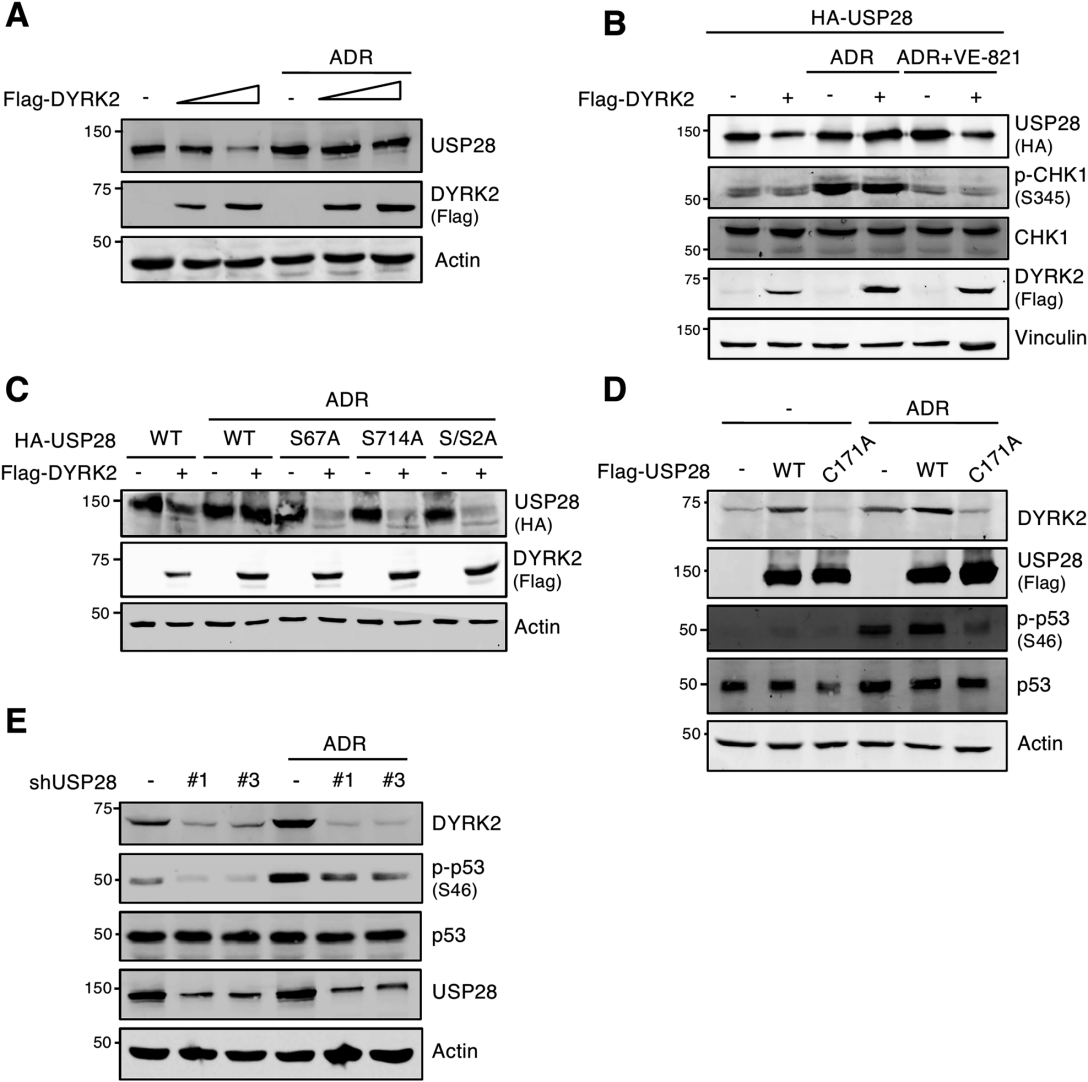
USP28 is required to DYRK2 stabilization in response to DNA damage. **A** HEK-293T cells were transfected with Flag-DYRK2 and treated or not with Adryamicin/Doxorrubicin (ADR) (3 μg/mL) for 12 h. USP28 levels were determined by WB. **B** HeLa cells were transfected with indicated plasmids and treated with the ATR inhibitor VE-821 (2.5  μM) and/or Adryamycin (ADR) (3 μg/mL) during 14 and 12 h, respectively. Phosphorylation of CHK1 at S345 was used as control for the treatments. USP28 degradation by DYRK2 was determined by WB. **C** HEK-293T cells were transfected with the indicated plasmids in presence or absence of Flag-DYRK2 WT and treated or not with Adryamicin/ Doxorrubicin (ADR) (3 μg/mL) for 12 h. HA-USP28 degradation by Flag-DYRK2 was followed by WB analysis. **D** HEK-293T cells were transfected with Flag-USP28 WT or an inactive mutant (Flag-USP28 C171A) treated or not with ADR (3 μg/mL) for 12 h and DYRK2 endogenous stabilization was analyzed by WB. **E** HEK-293T cells were transfected with two different USP28 shRNAs and treated or not with ADR (3 μg/mL) for 12 h. Protein levels were determined by WB using specific antibodies. Note: All experiments were performed at least 3 times, and a representative blot is shown.

Given the pivotal roles of USP28 and DYRK2 in the DNA damage response [10, 15], and the observed changes in their co-localization under these conditions (Fig. 6A), we investigated the impact of DNA damage on their bidirectional regulation. We first examined USP28 regulation by DYRK2 following exposure to the DNA-damaging agent Doxorubicin/Adriamycin (ADR). As shown in Figs. 7A and S7A, USP28 degradation induced by DYRK2, either WT or KM version, does not occur under DNA damage conditions. Previous studies have shown that USP28 is stabilized by ATM/ATR via phosphorylation at S67 and S714 upon DNA damage [10, 15]. To assess the functional relevance of these modifications, we analyzed the effects of ATR inhibition using VE-821. As shown in Fig. 7B, ATR inhibition restored USP28 degradation in the presence of ADR. Moreover, non-phosphorylatable USP28 mutants on the ATR/ATR residues responded to degradation by DYRK2 upon ADR treatment (Fig. 7C). Importantly, no differences were observed between USP28 and these mutants in either DYRK2-induced degradation without DNA damage (Fig. S7B) or their subcellular localization in response to DNA damage (Fig. S7C). These findings suggest that USP28 phosphorylation by ATR is critical for avoiding its degradation by DYRK2 under genotoxic stress.

To further elucidate the reciprocal regulatory axis between USP28 and DYRK2, we investigated the impact of DNA damage on USP28-dependent stabilization of DYRK2. As shown in Fig. 7D, DNA damage not only sustains but also enhances USP28-mediated stabilization of DYRK2. Notably, the catalytically inactive USP28 mutant (USP28^C171A^) significantly reduces basal DYRK2 stabilization under DNA damage, highlighting the requirement for USP28 enzymatic activity. Similar results were obtained with the ATR inhibitor (Fig. S7D) and ATM phosphorylation-deficient DYRK2 mutants [31] (Fig. S7E). USP28 mutants unable to be phosphorylated by ATR/ATM similarly stabilize DYRK2 as the wild-type version (Fig. S7F). These findings suggest that DYRK2 stabilization by USP28 is independent of ATM.

Since DYRK2 stabilization during DNA damage is involved in apoptosis via p53 S46 phosphorylation [31], we evaluated USP28 role in this signaling cascade. USP28 depletion using shRNA abolished DYRK2 stabilization following DNA damage, significantly reducing p53 phosphorylation at S46 (Fig. 7E). These data establish that USP28 is essential for stabilizing DYRK2 and enabling DYRK2-dependent p53 pro-apoptotic activation via S46 phosphorylation during the DNA damage response. This mechanism highlights USP28 as a critical node linking DNA damage signaling to apoptosis through DYRK2 regulation, emphasizing its role in maintaining genomic stability.

### Functional interplay between USP28 and DYRK2 in the DNA damage response

**Fig. 8:**
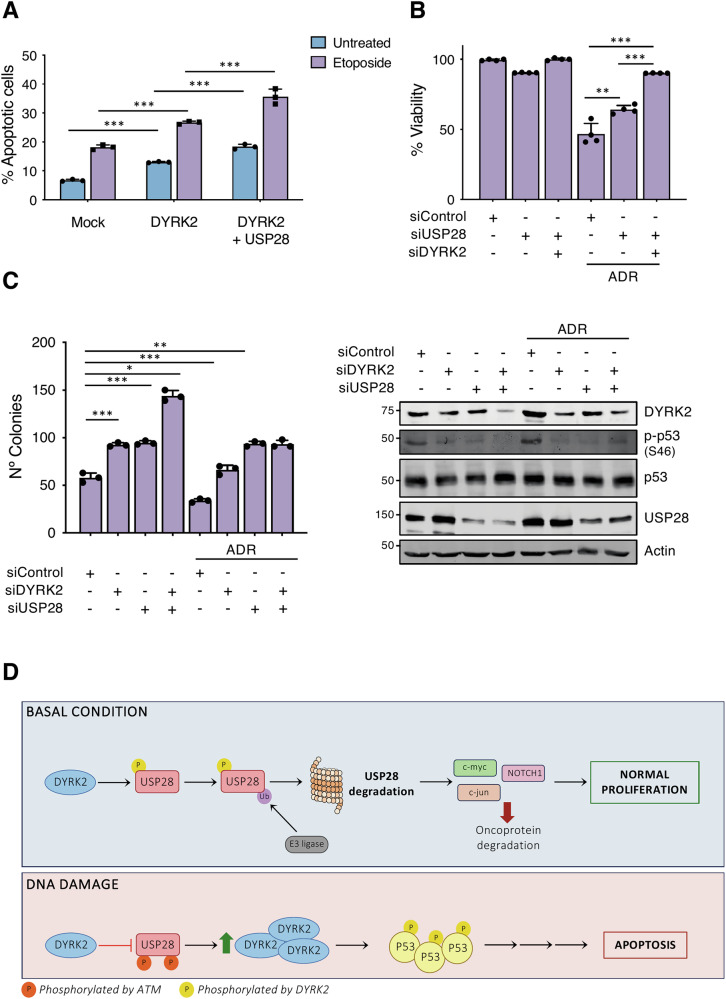
Functional interplay between USP28 and DYRK2 in the DNA damage response. **A** A549 cells were transfected and treated with etoposide (10 μM) for 24 h. Apoptosis was measured by flow cytometry with the Annexin V assay. **B** A549 cells were transfected with the indicated siRNAs and exposed or not to ADR (2 μg/mL) for 24 h. Viability was measured by the MTT assay. **C** A549 cells transfected with the indicated siRNA and exposed to ADR (2 μg/mL) for 24 h were subjected to a colony formation assay. The level of the indicated proteins in parallel samples was analyzed by WB to determine siRNA efficacy. Data represents mean ± SD of 3 different biological replicates (*P < 0.05; **P < 0.01, ***P < 0.001). **D** Model summarizing the results.

As shown in the previous sections, both DYRK2 and USP28 increase their expression and activity in response to genotoxic stress and DNA damage leading to p53 pro-apoptotic phosphorylation. To further investigate their functional interaction, we first analyzed their effects on apoptosis in lung cancer cells, given the correlation observed between these two proteins in this type of tumor (Fig. 1H, I). Apoptosis assays in A549 cells treated with etoposide (ETP) revealed that ectopic DYRK2 expression enhanced apoptosis, an effect that was further amplified by USP28 co-expression (Fig. 8A). Similar results were obtained in other lung cancer cell lines such as A427 and H460 (Fig. S8A). Conversely, USP28 depletion conferred resistance to ADR, a phenotype exacerbated by the simultaneous depletion of DYRK2 (Fig. 8B).

To investigate the pro-cytotoxic role of the DYRK2 and USP28 crosstalk in response to genotoxic stress, we used clonogenic assays. Knockdown of either DYRK2 or USP28 in A549 cells significantly increased their clonogenic capacity, with the simultaneous silencing of the two proteins enhancing the effect (Fig. 8C). Notably, in the absence of ADR, USP28 co-depletion enhanced DYRK2 pro-apoptotic effect, suggesting an inhibitory role in DYRK2-mediated apoptosis. This implies that DYRK2 activity may depend on USP28. However, under ADR treatment, this effect was abolished, supporting our previous findings that DYRK2 pro-apoptotic function is modulated by USP28 under basal conditions but becomes independent of USP28 in response to oncogenic stress. Collectively, these findings underscore the critical interplay between DYRK2 and USP28 in regulating cellular survival under genotoxic stress.

## Discussion

In this study, we have identified a novel bidirectional regulatory mechanism between the protein kinase DYRK2 and the deubiquitinase USP28, two key proteins involved in DNA damage response and cellular homeostasis. Our findings demonstrate that DYRK2 phosphorylates USP28, leading to its ubiquitination and proteasomal degradation, while USP28 acts stabilizing DYRK2 protein levels and kinase activity by its deubiquitination. This interaction suggests a finely tuned feedback loop that integrates DNA damage response pathways and cellular stress signaling (Fig. 8D).

Our results demonstrate that DYRK2 negatively regulates USP28 protein levels independently of its kinase activity, promoting its degradation via the ubiquitin-proteasome system. Similar activity-independent functions have been described for other kinases [32]. DYRK2 role as a scaffold for the EVDP complex is crucial for degrading key proteins in cell division, such as Katanin/p60 or TET [17, 25]. The ability of a DYRK2 truncated mutant, unable to interact with EVDP [17], to still promote USP28 degradation suggests DYRK2 may scaffold other unidentified E3 ligase complexes.

Our results show that while DYRK2 acts as a scaffold to facilitate USP28 ubiquitination, it also directly phosphorylates USP28, affecting its regulation. DYRK2 phosphorylates at least 24 residues, including S81 (between the UBA and SIM domains) and six within the catalytic domain (S248, S375, S504, S521, S532, S641). S375, S504, S521, and S532 are absent in USP25, possibly explaining regulatory differences (Fig. S2E). Nine phosphorylation sites were identified in the C-terminal region, with the T516/S517/S518 cluster being crucial for DYRK2-induced USP28 degradation, as mutations prevented its destabilization. However, USP28 may have unknown functional outputs as a DYRK2 substrate, with catalytic and non-catalytic functions converging in its regulation.

Notably, our findings suggest that DYRK2 kinase activity and its scaffold function are not independent mechanisms, but rather interrelated processes that may influence each other. Supporting this notion, the kinase-dead DYRK2 mutant displays increased affinity for USP28 and promotes its degradation more efficiently than the wild-type protein (Fig. S6A). Moreover, we observed that the interaction between DYRK2 and USP28 is modulated by the phosphorylation status of USP28: the triple phospho-deficient mutant exhibits reduced binding to DYRK2, whereas a phosphomimetic USP28 mutant (T516D/S517D/S518D) shows enhanced interaction (Fig. S2G). These results point to a phosphorylation-dependent regulation of DYRK2 scaffold activity and substrate’s affinity, underscoring the complexity of the mechanisms governing DYRK2-mediated signaling. Nevertheless, further investigation will be required to fully dissect this regulatory axis, which remains experimentally challenging to resolve.

From the perspective of this bidirectional regulation, we show that USP28 stabilizes DYRK2 by directly deubiquitinating it. USP28 overexpression increases DYRK2 protein levels without affecting transcription, while its reduction or inhibition decreases DYRK2 stability. This requires the deubiquitinase activity of USP28, as a catalytically inactive mutant fails to stabilize DYRK2. Accordingly, USP28 depletion increases DYRK2 ubiquitination, leading to its degradation. These findings identify USP28 as the first known DYRK2 deubiquitinase, acting as a regulatory factor that maintains DYRK2 stability and activity under DNA damage response.

Notably, we show that T525 is crucial for USP28-mediated DYRK2 stabilization, suggesting phosphorylation may facilitate this process. DYRK2 autophosphorylation is excluded, as DYRK2 kinase-deficient mutant are also stabilized by USP28. This effect increases after DNA damage, implying involvement of a DNA damage-activated kinase. ATM, known to phosphorylate DYRK2 on T33A and S369A [31], could target other residues, but T525 does not match its consensus sequence, and USP28-mediated stabilization persists under ATM inhibition. This suggests involvement of other, possibly proline-directed, kinases. Additionally, non-lysine deubiquitination should be considered, as many DUBs remove ubiquitin from threonine [33].

DYRK2 and USP28 regulate the DNA damage response [10, 22, 34], and their interaction may act as a molecular switch in cell fate under genotoxic stress. Our results show that, under these conditions, ATM/ATR-mediated phosphorylation of USP28 at S67 and S714 [10, 15] protects it from DYRK2-mediated degradation, allowing USP28 to stabilize DYRK2 and promote p53 phosphorylation at S46, crucial for apoptosis [35]. This mechanism underscores the importance of USP28 in linking DNA damage signaling to apoptotic outcomes.

USP28, FBXW7, and DYRK2 regulate proto-oncoproteins like c-Jun, c-Myc, NOTCH1, mTOR, and SNAIL [21, 23, 36–44]. DYRK2 phosphorylates these substrates, facilitating FBXW7-mediated degradation [16, 24], while USP28 deubiquitinates them, balancing stability. USP28 also stabilizes FBXW7, enhancing substrate degradation [26]. Additionally, DYRK2 phosphorylates and promotes FBXW7 [24] and USP28 degradation, exerting opposing effects. Our study shows that DYRK2 drives FBXW7 degradation under DNA damage, while USP28 degradation is inhibited. This aligns with previous findings: FBXW7 promotes p53 degradation and cell cycle re-entry [45], whereas USP28 and DYRK2 contribute to apoptosis via p53 deubiquitination or FBXW7 degradation and S46 phosphorylation, respectively [22, 34].

The functional consequence of this regulation is evident in apoptosis assays, where DYRK2-mediated phosphorylation of p53 at S46 is significantly reduced upon USP28 depletion. Since this phosphorylation promotes apoptosis, USP28 may enhance DYRK2 pro-apoptotic function under DNA damage. Supporting this hypothesis, cells lacking both DYRK2 and USP28 are more resistant to genotoxic stress, underscoring the importance of this regulatory axis in cell fate. These findings highlight its dynamic, context-dependent role in survival and its potential for developing therapies targeting genomic instability, particularly in resistant tumors. Enhancing DYRK2 activity could sensitize tumors with FBXW7 mutations by promoting USP28 degradation and preserving p53.

In conclusion, our study reveals a bidirectional regulatory mechanism between DYRK2 and USP28, integrating DNA damage response and cellular proto-oncoprotein homeostasis. This intricate interplay influences key apoptotic and oncogenic pathways, particularly through p53 regulation. Understanding this dynamic regulation could provide new therapeutic strategies for targeting tumors with genomic instability.

## Supplementary information

SUPPLEMENTAL MATERIAL

Supplementary original blots

## Data Availability

All data generated or analysed during this study are included in this published article and its supplementary information files.
