# Supplementary material for: A novel feedback loop between DYRK2 and USP28 regulates cancer homeostasis and DNA damage signaling
Source: Cell Death Differ. 2025 Aug 26;33(1):77–91. doi: 10.1038/s41418-025-01565-w (PMC12811399; doi:10.1038/s41418-025-01565-w)

Supplemental Material for:

A Novel Feedback Loop Between DYRK2 and USP28 Regulates Cancer Homeostasis and DNA Damage Signaling

Lucía Suanes-Cobos[1,2,3], Irene Aguilera Ventura[1,2,3], Miguel Torres Ramos[1,2,3], Alejandra Serrano-Yubero[1,2,3], Claudia Moreno Fernández-Aliseda[1,2,3], Silvia Fernández Álvarez [1,2,3], Martin Garrido-Rodríguez [4,5], Susana de la Luna [6,7,8], Cristian Prieto-Garcia [9,10], Markus E Diefenbacher[11,12,13], Ernesto Mejías-Pérez[1,2,3] and Marco A. Calzado[1,2,3]

[1] Instituto Maimónides de Investigación Biomédica de Córdoba (IMIBIC), Córdoba, Spain.
[2] Departamento de Biología Celular, Fisiología e Inmunología, Universidad de Córdoba, Córdoba, Spain.
[3] Hospital Universitario Reina Sofía, Córdoba, Spain.
[4] Genome Biology Unit, European Molecular Biology Laboratory, Heidelberg, Germany.
[5] Institute for Computational Biomedicine, Heidelberg University and Heidelberg University Hospital, Heidelberg, Germany.
[6] Centre for Genomic Regulation (CRG), The Barcelona Institute of Science and Technology (BIST), 08003, Barcelona, Spain.
[7] Centro de Investigación Biomédica en Red en Enfermedades Raras (CIBERER), Barcelona, Spain.
[8] Institució Catalana de Recerca i Estudis Avançats (ICREA), 08010, Barcelona, Spain.
[9] Protein Stability and Cancer Group, Department of Biochemistry and Molecular Biology, Theodor-Boveri-Institute, Biocenter, Am Hubland, 97074, Würzburg, Germany.
[10] Institute of Biochemistry II, Goethe University Frankfurt, Theodor-Stern-Kai 7, Haus 75, 60590, Frankfurt am Main, Germany.
[11] Institute of Lung Health and Immunity, Helmholtz Center Munich, Germany and German Center for Lung Research, DZL, Germany
[12] Ludwig Maximilian University Munich, Germany
[13] DKTK Munich, Germany

1. Supplemental Methods

mRNA extraction and RT-qPCR

Cells were washed twice in PBS and mRNA was extracted using High Pure RNA Isolation kit. Subsequently, mRNA was converted to cDNA employing iScript cDNA Synthesis kit. Finally, qPCR was performed using the fluorescent iQTM SYBR Green Supermix in an iCYCLER machine. The data of the gene of interest were normalized based on the housekeeping gene *HPRT* and gene expression was calculated using $2^{-\Delta\Delta Ct}$. The primers and commercial kits used are described in Supplemental Table 2.

Immunofluorescence

CHO cells were seeded on glass overlaps, transfected and treated with MG-132 together with/ without etoposide. After 48 hours of transfection, cells were fixed with 4% paraformaldehyde/PBS, permeabilized with 0,1% Triton-X100/PBS, blocked with BSA/PBS for 1 hour and incubated with specific primary antibodies overnight. Then, cells were washed with PBS three times and incubated for 1 hour at RT with secondary antibodies. After another three times PBS washes, cells were mounted on glass slides employing mounting-medium containing DAPI. Images were determined by confocal microscopy using LSM 5 EXCITER (Carl Zeiss MicroImaging GmbH, Oberkochen, Germany) together with ZEN 2008 software (Carl Zeiss MicroImaging GmbH). To analyze subcellular localization of USP28 and DYRK2 and its co-localization we employed Fiji and ImageJ software. RGB profile determined fluorescence intensity of each signal through white line.

***In vitro* kinase (IVK) and mass spectrometry (MS) analysis**

Bacterially expressed USP28 human recombinant protein was incubated while shaking for 1 hour at 30 °C with or without bacterially expressed DYRK2 human

recombinant protein, ATP and/or λ-phosphatase in kinase buffer. Then, samples were analyzed by WB or sent to CRG/UPF Proteomics Unit for MS analysis. Reagents and buffer composition are listed in Supplemental Table 2 and Supplemental Table 4.

For MS spectrometry analysis samples were analyzed using an LTQ-Orbitrap Fusion Lumos mass spectrometer (Thermo Fisher Scientific, San Jose, California, USA) coupled to an EASY-nLC 1 000 (Thermo Fisher Scientific (Proxeon), Odense, Denmark). Peptides were loaded directly onto the analytical column and were separated by reversed-phase chromatography using a 50-cm column with an inner diameter of 75 μm, packed with 2 μm C18 particles spectrometer (Thermo Scientific, San Jose, California, USA). Chromatographic gradients started at 95% buffer A and 5% buffer B with a flow rate of 300 nl/min for 5 min and gradually increased to 25% buffer B and 75% buffer A in 52 min and then to 40% buffer B and 60% buffer A in 8 min. After each analysis, the column was washed with 10% buffer A and 90% buffer B for 10 min. The mass spectrometer was operated in positive ionization mode with nanospray voltage set at 2.4 kV and source temperature at 275 °C. Ultramark 1621 was used for external calibration of the FT mass analyzer prior the analyses, and an internal calibration was performed using the background polysiloxane ion signal at m/z 445.1200. The acquisition was performed in data-dependent acquisition mode and full MS scans with 1 micro scan at a resolution of 120 000 were used over a mass range of m/z 350-1,500 with detection in the Orbitrap mass analyzer. Auto gain control was set to 1E5 and charge state filtering disqualifying singly charged peptides was activated. In each cycle of data-dependent acquisition analysis, following each survey scan, the most intense ions above a threshold ion count of 10 000 were selected for fragmentation. The number of selected precursor ions for fragmentation was

determined by the "Top Speed" acquisition algorithm and a dynamic exclusion of 60 s. Fragment ion spectra were produced via high-energy collision dissociation at normalized collision energy of 28% and they were acquired in the ion trap mass analyzer. AGC was set to 1E4, and an isolation window of 1.6 m/z and a maximum injection time of 200 ms were used. Digested BSA (New England Biolabs cat # P8108S) was analyzed between each sample to avoid sample carryover and to assure stability of the instrument and QCloud [1] was used to control instrument longitudinal performance.

Nucleus-Cytoplasm Subcellular fractionation

Cells washed with PBS were resuspended and incubated on ice with 500 μl of subcellular fractionation buffer A. After this time, 15 μl of 10% NP-40 was added, and the samples were gently vortexed and centrifuged for 10s at 16,100 xg. The cytoplasmic fraction (supernatant) was collected in a new tube. Nuclear pellet was washed with buffer A for 5 times, resuspended in subcellular fractionation buffer B, sonicated and centrifuged at 16,100 xg for 15 minutes. Finally, the supernatant corresponding to the nuclear fraction was collected.

Apoptosis, clonogenic and viability assays

For apoptosis assays, A549, H460 and A427 cells were collected in PBS. After resuspension in Annexin binding buffer, cells were stained with Annexin V and propidium iodide. Apoptosis was analyzed using a BD FACSCanto flow cytometer, with data acquisition analyzed using BD FACSDiva Software.

For clonogenic survival assay, A549 cells were transfected and seeded in 24-well plates. Afterward, cells were exposed to Adriamycin (2 μg/mL) for 24 hours. A total of 1000 treated cells were then plated in 6-well plates and allowed to grow for 10 days.

Then, cells were treated with Crystal violet solution (containing 6% glutaraldehyde and 0.5% W/V Crystal violet) for staining. Colonies, defined as groups of more than 50 cells, were counted and analyzed using Image J Software.

CPTAC data analysis

Harmonized proteomic and phospho-proteomic data from the Clinical Proteome Tumor Analysis Consortium (CPTAC) were obtained from the CPTAC Pan-Cancer Analysis Portal (https://proteomic.datacommons.cancer.gov/pdc/cptac-pancancer). Specifically, we retrieved the data tables preprocessed using the Broad Institute pipeline (*_Broad_Institute_harmonized.tsv.gz) and imported them into R (v4.3.2) for correlation analyses.

Protein structure modeling

USP28 structure was obtained from alpha-fold and its 3D structures were designed using ChimeraX program [2]. The representation of the protein characteristic domains and motifs has been designed following the color code indicated in a surface model. Residues identified by mass spectrometry have been colored in yellow. In the case of buried residues, these were colored in red in a stick model.

Two-dimensional (2D)-gel electrophoresis.

For each 2D-gel, 100 μg of total cell extracts were pre-cleaned using the methanol-chloroform protocol. Following this step, protein pellets were resuspended in lysis buffer (7M Urea, 2M Thiourea, 4% CHAPS and 0.002% bromophenol blue). A volume of 125 µL of each sample was then applied to IPG strips (ReadySTrip IPG Strips, 7cm, pH3-10, Cat #163200-BioRad) and placed into the focusing tray in (PROTEAN IEF CELL, BioRad, California, USA).

Samples were rehydrated for 12 hours at 20 °C, followed by isoelectric focusing at 4000V. IPG strips were incubated sequentially in equilibration buffer (6 M Urea, 2% SDS, 375 mM Tris-HCl pH 8,8, 20% glycerol): first with 2% DTT for 15 min, then with 2.5% iodoacetamide (VWR-LIFE SCIENCE, Ref. M216-30G) for an additional 15 min. Finally, proteins were resolved by SDS-PAGE.

BEAS-2B squamous-cell differentiation model

The human bronchial epithelial cell line BEAS-2B was obtained from SIGMA (ECACC, Salisbury, UK; catalog number 95102433) and cultured in LHC-9 medium (Life Technologies, Carlsbad, CA, USA), supplemented without serum, under standard conditions (37 °C, 5% CO_2, humidified atmosphere). To induce squamous differentiation, the cells were grown for 7 days in LHC-9 medium supplemented with 10% fetal bovine serum (FBS).

2. Supplemental References

1. Chiva C, Olivella R, Borras E, Espadas G, Pastor O, Sole A, et al. QCloud: A cloud-based quality control system for mass spectrometry-based proteomics laboratories. PLoS One. 2018;13:e0189209.

2. Goddard TD, Huang CC, Meng EC, Pettersen EF, Couch GS, Morris JH, et al. UCSF ChimeraX: Meeting modern challenges in visualization and analysis. Protein Sci. 2018;27:14-25.

3. Supplemental Figure Legends

Figure S1. DYRK2 regulates USP28 protein levels. (**A**) HeLa cells were transfected with a gradient of Flag-DYRK2 and Flag-USP28. Protein levels were determined by

WB. (**B**) HCT166 WT and FBXW7 knockout cells were transfected with Flag-DYRK2 wild-type (WT) or kinase-mutant version (KM) in presence of HA-USP28 and analyzed by WB. (**C**) HEK-293T cells were transfected with HA-USP28 with/without Flag-DYRK2 and/or a dominant-negative version of Cul1 (Flag-DN-Cul1). HA-USP28 protein level was analyzed by WB. Note: a representative blot from at least 3 biological replicates is shown.

Figure S2. DYRK2 phosphorylates USP28 promoting its degradation through a kinase activity-independent mechanism. (**A**) HEK-293T cells were transfected with HA-USP28 together with a gradient of wild-type or a kinase-mutant (KM) version of Flag-DYRK2. Protein levels were determined by WB. (**B**) HeLa cells were transfected with USP28 together with/without a gatekeeper mutant version of DYRK2 (GFP-DYRK2-GK). Cells were treated or not with PP1 Analog (3 μM) for 3 hours and protein levels determined by WB analysis. (**C**) USP28 protein sequence with the peptide coverage by MS/MS (~57%) after USP28 *in vitro* kinase assay with DYRK2. The peptides identified are shown in grey and the residues identified as phosphorylated are underlined in yellow. (**D**) 3D structure prediction of USP28 shown in a cartoon-atom model highlighting buried residues colored in red. Color code of USP28 structure is shown in Figure 2F. (**E**) Sequence alignment of human USP28 (isoform α, NP_065937), human USP25 (isoform α, NP_037528), and mouse USP28 (isoform 1, NP_780691) proteins performed using Clustal Omega (https://www.ebi.ac.uk/Tools/msa/clustalo/) with default settings. Protein domains are highlighted with boxes according to the color scheme in Figure 2F. Amino acids phosphorylated by DYRK2 are marked in red. (**F**), (**K**) HEK-293T cells were transfected with the indicated HA-USP28 mutants in presence or absent of Flag-

DYRK2. Protein levels were determined by WB. (**G**) Immunoprecipitation assays were done using a Flag antibody on lysates from HEK-293T cells transfected with either HA-USP28 WT, triple mutant T516A/S517A/S518A or T516D/S517D/S518D, in the presence or absence of Flag-DYRK2. Cells were treated with MG-132 (10 μM) for 12 hours before extract preparation. (**H**) HEK-293T cells were transfected with the HA-USP28 triple mutant T516A/S517A/S518A plasmid in the presence or absence of Flag-DYRK2. Total protein extracts were then treated or not with λ-phosphatase and subjected to 2D-gel separation. USP28 phosphorylation levels were analyzed by WB using an anti-HA antibody. (**I**) MDA-MB-468 DYRK2-knockout cells were transfected with the indicated plasmids and treated with MG-132 (10 μM) for 12 hours. USP28 Ser/Thr phosphorylation levels were analyzed by WB on the HA-USP28 immunoprecipitates. (**J**) HA-USP28 WT and triple mutant T516A/S517A/S518A immunoprecipitated from HEK-293T cells transfected with indicated plasmid were incubated in presence or absence of DYRK2 human recombinant protein, ATP and λ-phosphatase. Electrophoretic mobility was determined by WB. Changes in DYRK2 mobility are due to auto-phosphorylation. Note: all experiment were performed at least 3 times, and a representative blot is shown.

Figure S3. DYRK2 regulates USP28 via the ubiquitin-proteasome system. (**A**), (**B**) HEK-293T cells were transfected or not with HA-USP28 in the presence or absence of HA-DYRK2 and stimulated with cycloheximide (CHX; 20 μg/ml) for the indicated hours. Cells lysates were analyzed by WB. Data represent mean ± SD of HA-USP28 (**A**) or endogenous USP28 (**B**) normalized with vinculin, from 3 independent experiments (**P < 0.01; ***P < 0.001). (**C**) HEK-293T cells were transfected with HA-USP28 with/without Flag-DYRK2 wild-type (WT) or a kinase-

mutant version (KM). Cells were treated or not 12 hours with the proteasome inhibitor MG-132 (10 µM) prior to cell lysis for WB analysis. Note: we include a representative blot of more than 3 independent experiments.

Figure S4. USP28 stabilizes DYRK2 protein levels. (**A**) HEK-293T were transfected with Flag-DYRK2 together with a gradient of HA-USP28. Protein levels were determined by WB. (**B**) BEAS-2B cells differentiated to squamous phenotype in response to serum, A427, and H460 cells were transfected with HA-USP28, and protein levels were determined by WB. (**C**) Box plot showing DYRK2 and USP28 mRNA levels representing normalized counts from A431 cells stable expressing the indicated shRNAs (mean ± SD, n=3, ***P < 0.001). Data was obtained from public RNA-seq data (GSE129982). (**D**) HEK-293T cells were transfected with the indicated plasmids and cells lysates were analyzed by WB. Note: a representative blot is shown of at least three independent experiments.

Figure S5. USP28 deubiquitinates DYRK2. (**A**) HeLa cells were transfected with Flag-DYRK2 together with/without a gradient of wild-type or an inactive-mutant version (C171A, CA) of Flag-USP28. Protein levels were determined by WB. (**B**) HEK-293T cells were transfected with HA-DYRK2 in presence/absence of Flag-USP28 and treated or not with the USP28 inhibitor AZ1 (10 µM) for 24 hours. Cells lysates were studied by WB. (**C**) SK-MES-1 cells were transfected with wild-type or a deubiquitinase-mutant version (C171A, CA) of Flag-USP28. Protein levels were determined by WB. (**D**) HEK-293T cells were transfected with Flag-DYRK2 in presence or absence of HA-USP28 and treated with cycloheximide (CHX; 20 µg/mL) for the indicated times. Graph represents mean ± SD of Flag-DYRK2 levels normalized

by actin (***P < 0.001). Note: we show a representative result of at least three independent experiments.

Figure S6. DYRK2 interacts and co-localizes with USP28. (**A**) HEK-293T expressing HA-USP28 together with/without Flag-DYRK2 WT or kinase-deficient mutant (KM) and treated with the proteasome inhibitor MG-132 (10 µM) for 12 hours were used in an immunoprecipitation assay using Flag antibody. (**B**) Immunoprecipitation experiments performed using a HA antibody in HEK-293T cells lysates that were transfected with Flag-USP28 with/without wild-type (WT) or ΔC mutant version (lacking the 520-541 region) of HA-DYRK2 and treated with MG-132 (10 µM) for 12 hours. (**C**) HEK-293T cells transfected with the indicated plasmid were subjected to nucleus-cytoplasm fractionation and analyzed by WB. PPAR and Tubulin were used as markers of subcellular compartments. (**D**) HEK-293T cells transfected with the indicated Flag-DYRK2 versions in presence or absence of HA-USP28 were analyzed by WB. Note: we include a representative result of three independent experiments.

Figure S7. USP28 is required to DYRK2 stabilization in response to DNA damage. (**A**) HEK-293T cells were transfected with HA-USP28 in presence or absence of Flag-DYRK2 wild-type or kinase-mutant deficient (KM) and treated or not with Adriamycin/ Doxorrubicin (ADR) (3 µg/mL) for 12 hours. HA-USP28 and Flag-DYRK2 levels were determined by WB. (**B**) HEK-293T cells transfected with HA-USP28 wild-type (WT) or the indicated mutants in presence/absence of Flag-DYRK2 were analyzed by WB. (**C**) HEK-293T transfected with HA-USP28 or the indicated mutants and exposed to Adriamycin/Doxorrubicin (ADR) (3 µg/mL) for 12 hours were subjected to cytoplasm-nucleus subcellular fraction and analyzed by WB. (**D**) HEK-

293T transfected with Flag-DYRK2 with/without HA-USP28 were treated or not with Adriamycin/Doxorrubicin (ADR) (3 μg/mL) and/or the ATR inhibitor VE-821 (2.5 μM) for 12 hours or 14 hours, respectively. Cell lysates were analyzed by WB. (**E**) HEK-293T were transfected with DYRK2 wild-type or mutant versions that cannot be phosphorylated by ATM (T33A, T33A/S369A) in presence or absence of HA-USP28 and cell lysates analyzed by WB. (**F**) HEK-293T cells transfected with Flag-DYRK2 together with different version of HA-USP28 (wild-type or ATR non-phosphorylable USP28 mutants) were analyzed by WB.

Figure S8. Functional interplay between USP28 and DYRK2 in the DNA damage response. (**A**) A427 and H460 cells were transfected and treated with etoposide (10 μM) for 24 hours. Apoptosis was measured by flow cytometry with the Annexin V assay. Data represents mean ± SD of 3 different biological replicates (*P < 0.05; **P < 0.01, ***P < 0.001).

4. Supplemental Tables

Supplemental Table 1

Initial plasmid	Primers	Final plasmid
Flag-DYRK2 WT	F: 5'-GGGATCCTGCAGTGCGCATGGCCCCAGG-3'<br>R: 5'-GCAAAGCCTGGCCTGGGGCCATGCGCAC-3'	Flag-DYRK2 T525A
Flag-DYRK2 WT	F: 5'-GCTGAGGAGGCGGTTGCCAAGGCCTC-3'<br>R: 5'-CTCCCCGGTGGGAGGCCTTGGCAACC-3'	Flag-DYRK2 K541R
Flag-DYRK2 WT	F: 5' CTGCACTGTCACGACTCTCTGAGATGGCTC 3'<br>R: 5' GTTTAGGACCACAGAGCCATCTCAGAGAGTC 3'	Flag-DYRK2 S471X
Flag-DYRK2 WT	F: 5'-GATCCCCTTTTCCTTGACTTCTTAAGACAGTG-3'<br>R: 5'-GCAGGATCCCACTCTAAACACTGTCTTAAG-3'	Flag-DYRK2 K513R
Flag-DYRK2 T33A	F: 5'-GATTGAACTGTTGGGCATGCCCGCACAGAAACTG-3'<br>R: 5'-GATGCATCCAGCAGTTTCTGTGCGGGCATGCCCAAC-3'	Flag-DYRK2 T33A/S369A
HA-USP28 WT	F: 5'-CACTGATGAGAGAGTTAAGGAGCCCGCTCAAGACACTG-3'<br>R: 5'-GTTCTGTAGCAACAGTGTCTTGAGCGGGCTCCTTAAC-3'	HA-USP28 S67A
HA-USP28 WT	F: 5' GAACCATCTGAAGTAGAGGGGGCTGCTGCCAAC 3'<br>R: 5' CTTCCTTGTTGGCAGCAGCCCCCTCTACTTC 3'	HA-USP28 S81A
HA-USP28 WT	F: 5' GGATCTATTAAAGGGAGCATTCCGAGCAGCTGAGGAACAG 3'<br>R: 5' CTCACATCTTGCTGCTGTTCCTCAGCTGCTCGGAATGCTC 3'	HA-USP28 S248A/S249A
HA-USP28 WT	F: 5' GAACTCTCAAGATTTGAGTTTAATCAGGCCCTTGGG 3'<br>R: 5' GAATTTTCTCTGGCTGCCCAAGGGCCTGATTAAACTC 3'	HA-USP28 S375A
HA-USP28 WT	F: 5' CTCAGGATGTTGAAAGTACCTTTGCTGCTCCTGAAGATTC 3'<br>R: 5' GACTTGGGTAAAGAATCTTCAGGAGCAGCAAAGGTACTTTC 3'	HA-USP28 S503A/S504A
HA-USP28 WT	F: 5' GATTCTTTACCCAAGTCTAAACCACTGATGGAAATGCCTTC 3'<br>R: 5' GGAGCTGGCTGTGAAGGCATTTCCATCAGTGGTTTAGAC 3'	HA-USP28 Δ516-521
HA-USP28 WT	F: 5' CTCAGGATGTTGAAAGTACCTTTGCTGCTCCTGAAGATTC 3'<br>R: 5' GACTTGGGTAAAGAATCTTCAGGAGCAGCAAAGGTACTTTC 3'	HA-USP28 S503A-S504A
HA-USP28 WT	F: 5' GATTCTTTACCCAAGTCTAAACCACTGGCAGCTGCTCGGTCTTC 3'<br>R: 5' GAAGGCATTTCCATGGAAGACCGAGCAGCTGCCAGTGGTTTAG 3'	HA-USP28 T516A-S517A-S518A
HA-USP28 WT	F: 5' CTTTACCCAAGTCTAAACCACTGGATGATGATCGGTCTTCCATG 3'<br>R: 5' GAAGGCATTTCCATGGAAGACCGATCATCATCCAGTGGTTTAG 3'	HA-USP28 T516D-S517D-S518D
HA-USP28 WT	F: 5' GTCTAAACCACTGACATCTTCTCGGGCTGCCATGGAAATG 3'<br>R: 5' GCTGGCTGTGAAGGCATTTCCATGGCAGCCCGAGAAGATG 3'	HA-USP28 S520A-S521A
HA-USP28 WT	F: 5' CAAACTGCCCAGGCTATTGCAAACGCAGCCCG 3'<br>R: 5' CTTCTCATAGGCACGGGCTGCGTTTGCAATAG 3'	HA-USP28 T753A
HA-USP28 WT	F: 5' GGCTCTATCAGCTTGCCAAAGAGGCCCCCAC 3'<br>R: 5' GGATCACTGTGAGAGGTGGGGGCCTCTTTGG 3'	HA-USP28 T816A
HA-USP28 WT	F: 5' GCCGGGGGGTCAAAGAAGCCGTGATTGCTT 3'<br>R: 5' CGGTATAAAGCAATCACGGCTTCTTTGAC 3'	HA-USP28 S943A
HA-USP28 WT	F: 5'-GAGTCCTCCACCAACTCCTCAGCACAGGACTAC-3'<br>R: 5'-CTTGTGATGTAGAGTAGTCCTGTGCTGAGGAG-3'	HA-USP28 S714A
HA-USP28 S67A	F: 5'-GAGTCCTCCACCAACTCCTCAGCACAGGACTAC-3'<br>R: 5'-CTTGTGATGTAGAGTAGTCCTGTGCTGAGGAG-3'	HA-USP28 S67A/S714A
HA-USP28 WT	F: 5'-CTTTACCCAAGTCTAAACCACTGGCATCTTCTCG-3'<br>R: 5'-GCATTTCCATGGAAGACCGAGAAGATGCCAGTGG-3'	HA-USP28 T516A
HA-USP28 WT	F: 5'-CTTTACCCAAGTCTAAACCACTGACAGCTTCTCGG-3'	HA-USP28 S517A

	R: 5’-GAAGGCATTTCCATGGAAGACCGAGAAGCTGTCAG-3’	
HA-USP28 WT	F: 5’-CCCAAGTCTAAACCACTGACATCTGCTCGG-3’	HA-USP28 S518A
	R: 5’-GAAGGCATTTCCATGGAAGACCGAGCAGATG-3’	
HA-USP28 WT	F: 5’-GTCTAAACCACTGACATCTTCTCGGGCTTCCATGG-3’	HA-USP28 S520A
	R: 5’-GGCTGTGAAGGCATTTCCATGGAAGCCCGAGAAG-3’	
HA-USP28 WT	F: 5’-GTCTAAACCACTGACATCTTCTCGGTCTGCCATGG-3’	HA-USP28 S521A
	R: 5’-GAGCTGGCTGTGAAGGCATTTCCATGGCAGACCG-3’	

Supplemental Table 2

**Reagent or Resource**	**Source**	**Identifier**
**Antibodies**		
FLAG M2	Sigma-Aldrich	Cat#F3165<br>RRID: AB_259529
HA (3F10)	Roche Molecular Biochemicals	Cat#12158167001<br>RRID: AB_390915
Myc (9E10)	Roche Molecular Biochemicals	Cat#11667149001<br>RRID: AB_390912
GFP	Roche Molecular Biochemicals	Cat#11814460001<br>RRID: AB_390913
β-actin (AC-74)	Sigma-Aldrich	Cat#A2228<br>RRID: AB_476697
Vinculin (E1E9V)	Cell Signaling	Cat#13901<br>RRID: AB_2728768
Tubulin (DM1A)	Sigma-Aldrich	Cat#T9026<br>RRID: AB_477593
PPARg (C26H12)	Cell Signaling	Cat#2435<br>RRID: AB_2166051
DYRK2	Sigma-Aldrich	Cat#HPA027230<br>RRID: AB_1847925
USP28	Sigma-Aldrich	Cat#HPA006778<br>RRID: AB_1080520
USP25 (C-5)	Santa Cruz Biotechnology	Cat#sc-398414
p53 (DO-1)	Santa Cruz Biotechnology	Cat#sc-126<br>RRID: AB_628082
Phospho-p53-Ser46	ProteinTech	Cat#28960-1-AP<br>RRID: AB_2918223
Phospho-Chk1-Ser345 (133D3)	Cell Signaling	Cat#2348<br>RRID: AB_331212
CHK1 (2G1D5)	Cell Signaling	Cat#2360<br>RRID: AB_2080320
Jun (60A8)	Cell Signaling	Cat#9165<br>RRID: AB_2130165
HSF1	Enzo Life Science	Cat#ADI-SPA-901-D<br>RRID: AB_2039202
Phospho-HSF1-Ser 320	Abcam	Cat# ab76183<br>RRID: AB_1523789
Anti-phosphoserine/threonine	ECM Biosciences	Cat# PP2551<br>RRID: AB_1184778
StarBright ™ Blue 700 Goat Anti-Mouse IgG	Bio-Rad	Cat#12004159<br>RRID: AB_2884948
Goat anti Mouse IgG (H/L): DyLight®800	Bio-Rad	Cat# STAR117D800GA<br>RRID: AB_10845157
StarBright™ Blue 520 Goat Anti-Mouse IgG	Bio-Rad	Cat#12005866<br>RRID: AB_2934034
StarBright ™ Blue 700 Goat Anti-Rabbit IgG	Bio-Rad	Cat#12004161

		RRID: AB_2721073
StarBright ™ Blue 520 Goat Anti-Rabbit IgG	Bio-Rad	Cat#12005870<br>RRID: AB_2884949
Rabbit F(ab')2 anti Rat IgG: Dylight®800	Bio-Rad	Cat# STAR16D800GA RRID: AB_10842665
**Bacterial and virus strains**		
Escherichia coli TOP10	Thermo Fisher Scientific	Cat#C404010
XL 10-Gold Ultracompetent Cells	Agilent	Cat#200315
**Chemicals, peptides, and recombinant proteins**		
MG-132	Enzo Life Science	Cat#BML-PI102-0005
Cycloheximide	Sigma-Aldrich	Cat#C7698
Adriamycin/Doxorubicin	Sigma-Aldrich	Cat#44583
Etoposide	Sigma-Aldrich	Cat# E1383
1NM-PP1	SantaCruz Biotechnology	Cat# sc-203214
LDN192960	MedChemExpress	Cat# HY-13455
VE-821	MedChemExpress	Cat# HY-14731
USP28 [GST-tagged] recombinant protein	Ubiquigent	Cat#64-0020-050
DYRK2 recombinant protein	Merck	Cat# 14-669M
ATP	Sigma-Aldrich	Cat# A26209
Lambda Protein Phosphatase	New England Biolabs	Cat#P0753L
Dulbecco's Modified Eagle Medium high glucose, GlutaMAX™ Supplement	Gibco	Cat# 61965026
Eagle's Minimum Essential Medium	Gibco	Cat#15188319
McCoy's 5A (Modified) Medium	Gibco	Cat#16600082
Fetal Bovine Serum FBS	Gibco	Cat# 10270106
Trypsin-EDTA (0.25%), phenol red	Gibco	Cat# 25200072
Penicilin-Streptomycin	Sigma-Aldrich	Cat#P0781
Protein A/G Sepharose beads	Santa Cruz Biotechnology	Cat# sc-2003
Triton™ X-100	Sigma-Aldrich	Cat# T8787
Glutaraldehyde solution	Sigma-Aldrich	Cat# G6257
Paraformaldehyde	Sigma-Aldrich	Cat# 8.18715
Bovine Serum Albumin (BSA)	Sigma-Aldrich	Cat# A6003
DAPI (4',6-Diamidino-2-Phenylindole)	Thermo Fisher Scientific	Cat# D1306<br><br>RRID: AB_2629482
iQ™ SYBR® Green Supermix	Bio-Rad	Cat#1708880
cOmplete™ Protease Inhibitor Cocktail	Sigma-Aldrich	Cat# 11697498001
siRNA non-targeting pool	Dharmacon	Cat# D-001810-10-20
ON-TARGET plus SMARTpool against DYRK2	Dharmacon	Cat# L-004730-00-0020
Lipofectamine™ 2000 Transfection Reagent	Invitrogen	Cat#11668019
Polyethylenimine, Linear, MW 25000, Transfection Grade (PEI 25K™)	Polysciences	Cat# 23966
**Critical commercial assays**		
QuikChange Lightning Site-Directed Mutagenesis Kit	Agilent	Cat#210518
High Pure RNA Isolation kit	Roche Molecular Biochemicals	Cat#11828665001
iScript™ cDNA Synthesis Kit	Bio-Rad	Cat#1708890
**Experimental models: Cell lines**		
BEAS-2B	ATCC	CRL-3588
HEK293T	ATCC	CRL-3216
HCT116	ATCC	CCL-247
HCT-116 FBW7 -/-	Laboratory of Professor Bert Vogelstein	PMID:14999283
MDA-MB-468 WT and DYRK2 -/-	Laboratory of Laureano de la Vega	PMID: 31605148
CHO	ATCC	CCL-61
HeLa	ATCC	CCL-2
A549	ATCC	CCL-185

SK-MES-1	Laboratory of Professor Markus E. Diefenbacher	PMID: 34611298
**Oligonucleotides**		
Human USP28 realtime PCR primer forward: 5’ ACTCAGACTATTGAACAGATGTACTGC 3’		This study
Human USP28 realtime PCR primer reverse: 5’ CTGCATGCAAGCGATAAGG 3’		This study
Human DYRK2 realtime PCR primer forward: 5’ GTGGTCAAGGCCTACGATCACA 3’		This study
Human DYRK2 realtime PCR primer reverse: 5’ CCGCAGGTGTTCCAGGATTC 3’		This study
Human HPRT realtime PCR primer forward: 5’ AATTATGGACAGGACTGAACGTCTTGCT 3’		This study
Human HPRT realtime PCR primer reverse: 5’ TCCAGCAGGTCAGCAAAGAATTTATAGC 3’		This study
**Software and algorithms**		
Image Lab	BioRad	https://www.bio- rad.com/ en-us/product/image-labsoftware?ID=KRE 6P5E8Z
ImageJ v1.45	ImageJ	https://imagej.nih.gov/ij/
Fiji	Fiji	https://image.net/fiji
Prism7	GraphPad software	https://www.graphpad.com/scientific-software/prism/
ChimeraX	ChimeraX	https://www.rbvi.ucsf.edu/chimerax/

Supplemental Table 3

PLASMID	PROVIDER	SOURCE
HA-USP28	Markus E. Diefenbacher	PMID: 32128997
Flag-USP28	Markus E. Diefenbacher	PMID: 32128997
Flag-USP28 C171A	Markus E. Diefenbacher	PMID: 32128997
HA-USP25	Markus E. Diefenbacher	N/A
SH1-USP28	Markus E. Diefenbacher	PMID: 37202505
SH3-USP28	Markus E. Diefenbacher	PMID: 37202505
HA-DYRK1A	Susana de la Luna	PMID: 12799418
HA-DYRK1B	Susana de la Luna	PMID: 30979931
HA-DYRK2	Susana de la Luna	PMID: 21127067
HA-DYRK3	Susana de la Luna	PMID: 30979931
HA-DYRK4	Susana de la Luna	PMID: 21127067
Flag-DYRK2-cat	Susana de la Luna	N/A
Flag-DYRK2-ΔC	Susana de la Luna	N/A
HA-DYRK2-ΔC	Susana de la Luna	N/A
Flag-DYRK2 WT	Marco A. Calzado	PMID: 22878263
Flag-DYRK2 WT (SI)	Marco A. Calzado	PMID: 34363019
Flag-DYRK2 T33A	Marco A. Calzado	PMID: 34363019
Myc-DYRK2 WT	Marco A. Calzado	PMID: 22878263
GFP-DYRK2	Marco A. Calzado	PMID: 31605148
Flag-DYRK2 KM (K178M)	Marco A. Calzado	PMID: 31605148
HA-UBI	Marco A. Calzado	PMID: 34363019
GFP-DYRK2-GK	Laureano de la Vega	PMID: 33268814
Flag-dn-CUL1	Addgene (Cat #15818)	PMID: 16338364

Supplemental Table 4

BUFFER	COMPONENTS

IP Buffer	50 mM Hepes pH 7.5, 50 mM NaCl and 1% Triton X-100 supplemented with 5 mM EGTA, 20 mM $Na_4P_2O_7$, 50 mM NaF, 1 mM Na_3VO_4, 2 mM PMSF and cOmplete™ Protease Inhibitor Cocktail
NP40 buffer	50 mM Tris-HCl pH 7.5, 150 mM NaCl, 1% [v/v] NP-40, 10% [v/v] glycerol, 10 mM NaF, 1 mM Na_3VO_4, cOmplete™ Protease Inhibitor Cocktail and 1 mM PMSF
Apoptosis buffer	10 mM Hepes pH 7.4, 140 mM NaCl, 2.5 mM $CaCl_2$
Kinase buffer	20 mM Hepes pH 7.5, 10 mM $MgCl_2$, 1 mM DTT
Crystal violet	6% glutaraldehyde, 0.5% [w/v] Crystal violet
LC-MS buffer A	0.1% formic acid in water
LC-MS buffer B	0.1% formic acid in 80% CAN
Subcellular Fractionation buffer A	10 mM Hepes/KOH pH 7.9, 10 mM KCl, 0.1 mM EDTA, 0.1 mM EGTA, 1 mM β-Mercaptoethanol, 10 mM NaF, 1 mM Na_3VO_4, cOmplete™ Protease Inhibitor Cocktail and 1 mM PMSF
Subcellular Fractionation buffer B	20 mM Hepes/KOH pH 7.9, 400 mM NaCl, 1 mM EDTA, 1 mM EGTA, 1 mM β-Mercaptoethanol, 10 mM NaF, 1 mM Na_3VO_4, cOmplete™ Protease Inhibitor Cocktail and 1 mM PMSF

Supp. Figure 1

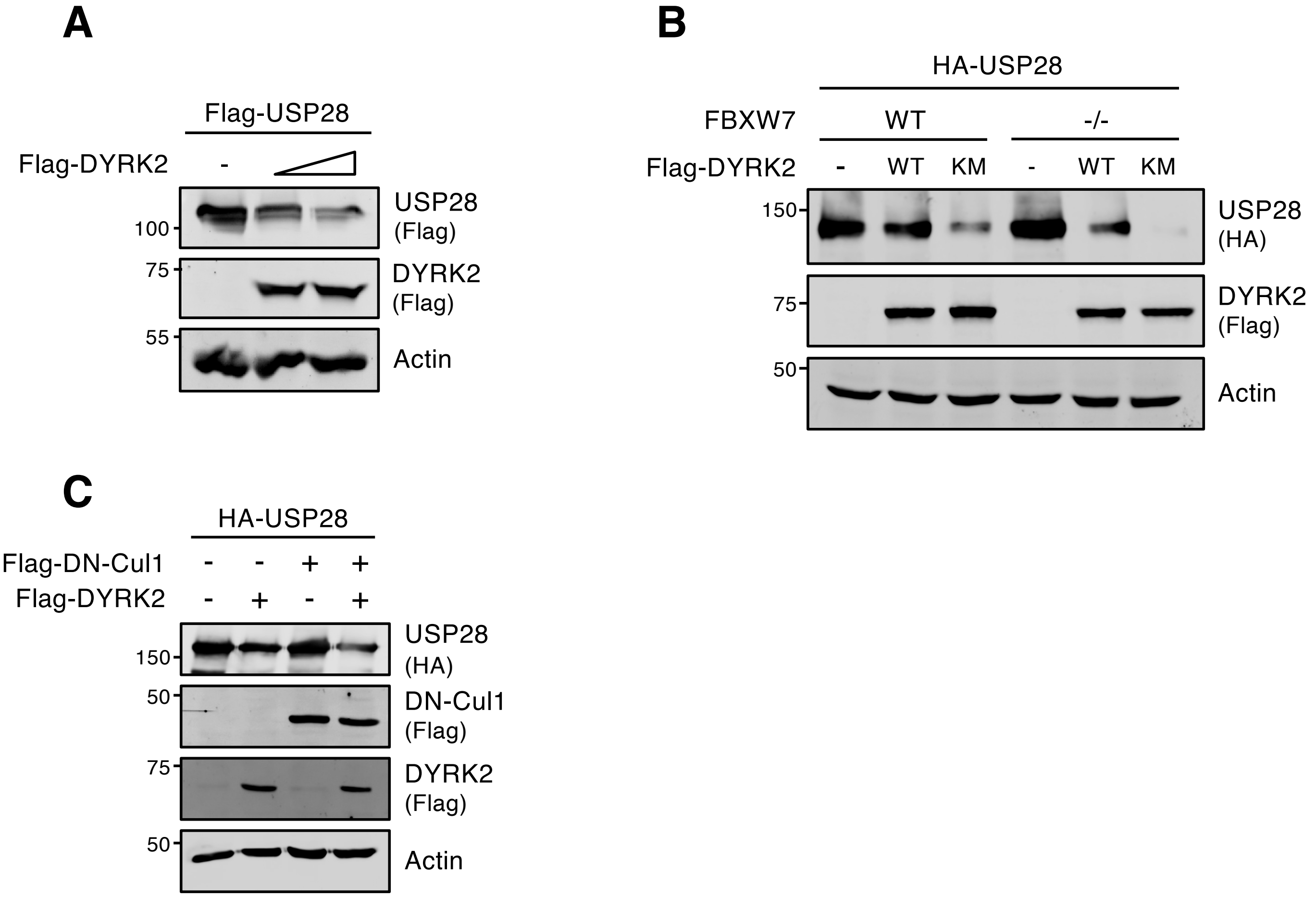

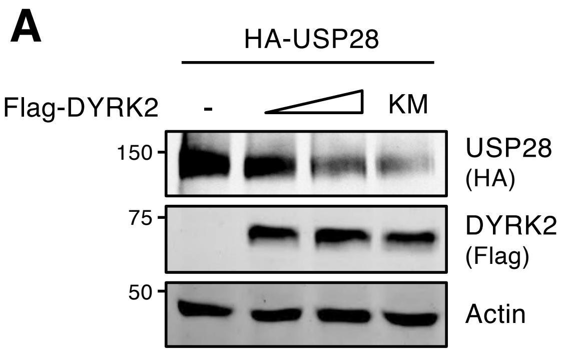

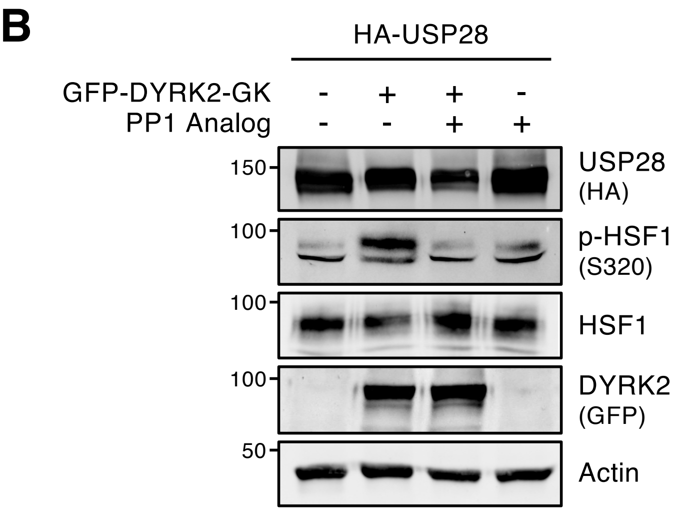

C

```
         10          20          30          40          50          60          70          80
MTAELQQDDA  AGAADGHGSS  CQMLLNQLRE  ITGIQDPSFL  HEALKASNGD  ITQAVSLLTD  ERVKEPSQDT  VATEPSEVEG
         90         100         110         120         130         140         150         160
SAANKEVLAK  VIDLTHDNKD  DLQAAIALSL  LESPKIQADG  RDLNRMHEAT  SAETKRSKRK  RCEVWGENPN  PNDWRRVDGW
        170         180         190         200         210         220         230         240
PVGLKNVGNT  CWFSAVIQSL  FQLPEFRRLV  LSYSLPQNVL  ENCRSHTEKR  NIMFMQELQY  LFALMMGSNR  KFVDPSAALD
        250         260         270         280         290         300         310         320
LLKGAFRSSE  EQQQDVSEFT  HKLLDWLEDA  FQLAVNVNSP  RNKSENPMVQ  LFYGTFLTEG  VREGKPFCNN  ETFGQYPLQV
        330         340         350         360         370         380         390         400
NGYRNLDECL  EGAMVEGDVE  LLPSDHSVKY  GQERWFTKLP  PVLTFELSRF  EFNQSLGQPE  KIHNKLEFPQ  IIYMDRYMYR
        410         420         430         440         450         460         470         480
SKELIRNKRE  CIRKLKEEIK  ILQQKLERYV  KYGSGPARFP  LPDMLKYVIE  FASTKPASES  CPPESDTHMT  LPLSSVHCSV
        490         500         510         520         530         540         550         560
SDQTSKESTS  TESSSQDVES  TFSSPEDSLP  KSKPLTSSRS  SMEMPSQPAP  RTVTDEEINF  VKTCLQRWRS  EIEQDIQDLK
        570         580         590         600         610         620         630         640
TCIASTTQTI  EQMYCDPLLR  QVPYRLHAVL  VHEGQANAGH  YWAYIYNQPR  QSWLKYNDIS  VTESSWEEVE  RDSYGGLRNV
        650         660         670         680         690         700         710         720
SAYCLMYIND  KLPYFNAEAA  PTESDQMSEV  EALSVELKHY  IQEDNWRFEQ  EVEEWEEEQS  CKIPQMESST  NSSSQDYSTS
        730         740         750         760         770         780         790         800
QEPSVASSHG  VRCLSSEHAV  IVKEQTAQAI  ANTARAYEKS  GVEAALSEVM  LSPAMQGVIL  AIAKARQTFD  RDGSEAGLIK
        810         820         830         840         850         860         870         880
AFHEEYSRLY  QLAKETPTSH  SDPRLQHVLV  YFFQNEAPKR  VVERTLLEQF  ADKNLSYDER  SISIMKVAQA  KLKEIGPDDM
        890         900         910         920         930         940         950         960
NMEEYKKWHE  DYSLFRKVSV  YLLTGLELYQ  KGKYQEALSY  LVYAYQSNAA  LLMKGPRRGV  KESVIALYRR  KCLLELNAKA
        970         980         990        1000        1010        1020        1030        1040
ASLFETNDDH  SVTEGINVMN  ELIIPCIHLI  INNDISKDDL  DAIEVMRNHW  CSYLGQDIAE  NLQLCLGEFL  PRLLDPSAEI
       1050        1060        1070
IVLKEPPTIR  PNSPYDLCSR  FAAVMESIQG  VSTVTVK
```

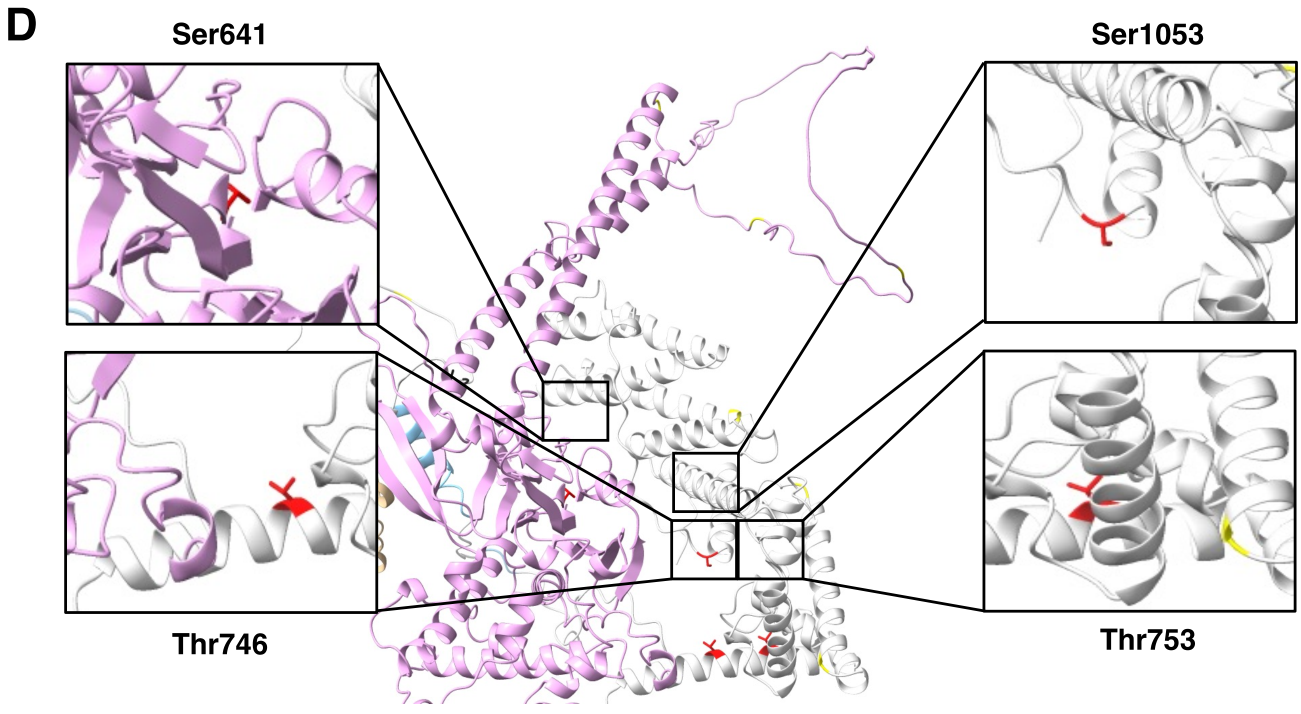

Supp. Figure 2

E

```
                                           UBA
USP28_Human   MTAELQQDDAAGAADGHGSSCQMLLNQLREITGIQDPSFLHEALKASNGDITQAVSLLTD   60
USP28_Mouse   MTAELQQDDSAGAADGHGSSCQMLLNQLREITGIQDPSFLHEALKASNGDITQAVSLLTD   60
USP25_Human   MTVEQ----NVLQQSAAQKHQQTFLNQLREITGINDTQILQQALKDSNGNLELAVAFLTA   56

                        S81            SIM       UIM
USP28_Human   ERVKEPSQDTVATEPSEVEGS----AANKEVLAKVIDLTHDNKDDLQAAIALSLLESPKI   116
USP28_Mouse   QRVKEPSHDTTAAEPSEVEES----ATSKDLLAKVIDLTHDNKDDLQAAIALSLLESPNI   116
USP25_Human   KNAKTPQQEETTYYQTALPGNDRYISVGSQADTNVIDLTGDDKDDLQRAIALSLAESNRA   116

                                               Catalytic domain
USP28_Human   QA------DGRDLNRMHEATSAET-KRSKRKRCEVWGENPNPNDWRRVDGWPVGLKNVGN   169
USP28_Mouse   QA------DNRDLNRAHEANSAET-KRSKRKRCEVWGENHNPNNWRRVDGWPVGLKNVGN   169
USP25_Human   FRETGITDEEQAISRVLEASIAENKACLKRTPTEVWRDSRNPYDRKRQDKAPVGLKNVGN   176

USP28_Human   TCWFSAVIQSLFQLPEFRRLVLSYSLPQNVLENCRSHTEKRNIMFMQELQYLFALMMGSN   229
USP28_Mouse   TCWFSAVIQSLFQLPEFRRLVLSYNLPQNILENCRSHTEKRNIMFMQELQYLFALLLGSN   229
USP25_Human   TCWFSAVIQSLFNLLEFRRLVLNYKPPSNAQDLPRNQKEHRNLPFMRELRYLFALLVGTK   236

                          S248/S249
USP28_Human   RKFVDPSAALDLLKGAFRSSEEQQQDVSEFTHKLLDWLEDAFQLAVNVNS-PRNKSENPM   288
USP28_Mouse   RKFVDPSAALDLLKGAFRSSEEQQQDVSEFTHKLLDWLEDAFQLAVNVNSHLRNKSENPM   289
USP25_Human   RKYVDPSRAVEILKDAFKSNDSQQQDVSEFTHKLLDWLEDAFQMKAEEET-DEEKPKNPM   295

USP28_Human   VQLFYGTFLTEGVREGKPFCNNETFGQYPLQVNGYRNLDECLEGAMVEGDVELLPSDHSV   348
USP28_Mouse   VQLFYGTFLTEGVREGKPFCNNETFGQYPLQVNGYHNLDECLEGAMVEGDIALLPSDRSV   349
USP25_Human   VELFYGRFLAVGVLEGKKFENTEMFGQYPLQVNGFKDLHECLEAAMIEGEIESLHSENSG   355

                                S375
USP28_Human   KYGQERWFTKLPPVLTFELSRFEFNQSLGQPEKIHNKLEFPQIIYMDRYMYRSKELIRNK   408
USP28_Mouse   KYGQERWFTKLPPVLTFELSRFEFNQSLGQPEKIHNKLEFPQIIYMDRYMYKSKELIRSK   409
USP25_Human   KSGQEHWFTELPPVLTFELSRFEFNQALGRPEKIHNKLEFPQVLYLDRYMHRNREITRIK   415

USP28_Human   RECIRKLKEEIKILQQKLERYVKYGSGPARFPLPDMLKYVIEFASTKPASESCPPESDTH   468
USP28_Mouse   RESVRKLKEEIQVLQQKLERYVKYGSGPSRFPLPDMLKYVIEFASTKPASESCLSGSAEH   469
USP25_Human   REEIKRLKDYLTVLQQRLERYLSYGSGPKRFPLVDVLQYALEFASSKPVCTSPVDDIDAS   475
                                                             T516/S517/S518
                                            S503/S504         S520/S521
USP28_Human   MTLPLSSVHCSVSDQTSKESTSTESSSQDVESTFSSPEDSLPKSK----PLTSSRSSMEM   524
USP25_Human   VTLPLPSVHCPISDLTPKESSSPESCSQNAGSTFSSPEDALPSSEGMNGPFTSPHSSLET   529
USP25_Human   S-PPSGSI--PSQT-LPSTTEQQGALSSELPSTSPSSVAAISSRSVIHKPFTQSRIPPDL   531

                 T532
USP28_Human   PSQPAPRTVTDEEINFVKTCLQRWRSEIEQDIQDLKTCIASTTQTIEQMYCDPLLRQVPY   584
USP28_Mouse   PAPPAPRTVTDEEMNFVKTCLQRWRSEIEQDIQDLKNCISSSTKAIEQMYCDPLLRQVPY   589
USP25_Human   PMHPAPRHITEEELSVLESCLHRWRTEIENDTRDLQESISRIHRTIELMYSDKSMIQVPY   591

                                                                   S641
USP28_Human   RLHAVLVHEGQANAGHYWAYIYNQPRQSWLKYNDISVTESSWEEVERDSYGGLRNVSAYC   644
USP28_Mouse   RLHAVLVHEGQASAGHYWAYIYNQPRQTWLKYNDISVTESSWEELERDSYGGLRNVSAYC   649
USP25_Human   RLHAVLVHEGQANAGHYWAYIFDHRESRWMKYNDIAVTKSSWEELVRDSFGGYRNASAYC   651

USP28_Human   LMYINDKLPYFNAEAAPTESDQ-MSEVEALSVELKHYIQEDNWRFEQEVEEWEEEQSCKI   703
USP28_Mouse   LMYINDNLPHFSAEASSNESDETAGEVEALSVELRQYIQEDNWRFQQEVEEWEEEQSCKI   709
USP25_Human   LMYINDKAQFLIQEEFNKETGQPLVGIETLPPDLRDFVEEDNQRFEKELEEWDAQLAQKA   711

                S709/T710     S724/S727/S728     T746     T753
USP28_Human   PQMESSTNSSSQDYSTSQEPSVASSHGVRCLSSEHAVIVKEQTAQAIANTARAYEKSGVE   763
USP28_Mouse   PQMESSPNSSSQDFSTSQESPAVSSHEVRCLSSEHAVIAKEQTAQAIANTAHAYEKSGVE   769
USP25_Human   LQEKLLASQKLRESETSVT----------------------TAQAAG--DPEYLEQPSR   746

                                     T794                 T816
USP28_Human   AALSEVMLSPAMQGVILAIAKARQTFDRDGSEAGLIKAFHEEYSRLYQLAKETPTSHSDP   823
USP28_Mouse   AAL--------------------------------SEAFHEEYSRLYQLAKETPTSHSDP   797
USP25_Human   SDFSKH----LKEETIQIITKASHEHEDKSPETVLQSAIKLEYARLVKLAQEDTPPETDY   802

USP28_Human   RLQHVLVYFFQNEAPKRVVERTLLEQFADKNLSYDERSISIMKVAQAKLKEIGPDDMNME   883
USP28_Mouse   RLQHVLVYFFQNEAPKRVVERTLLEQFADRNLSYDERSISIMKVAQAKLMEIGPDDMNME   857
USP25_Human   RLHHVVVYFIQNQAPKKIIEKTLLEQFGDRNLSFDERCHNIMKVAQAKLEMIKPEEVNLE   862

                                                                    S943
USP28_Human   EYKKWHEDYSLFRKVSVYLLTGLELYQKGKYQEALSYLVYAYQSNAALLMKGPRRGVKES   943
USP28_Mouse   EYKRWHEDYSLFRKVSVYLLTGLELFQKGKYQEALSYLVYAYQSNAGLLVKGPRRGVKES   917
USP25_Human   EYEEWHQDYRKFRETTMYLIIGLENFQRESYIDSLLFLICAYQNNKELLSKGLYRGHDEE   922

USP28_Human   VIALYRRKCLLELNAKAASLFETNDDHSVTEGINVMNELIIPCIHLIINNDISKDDLDAI   1003
USP28_Mouse   VIALYRRKCLLELNAKAASLFETNDDHSVTEGINVMNELIIPCIHLIINNDISKDDLDAI   977
USP25_Human   LISHYRRECLLKLNEQAAELFESGEDREVNNGLIIMNEFIVPFLPLLLVDEMEEKDILAV   982

                                                        S1053
USP28_Human   EVMRNHWCSYLGQDIAENLQLCLGEFLPRLLDPSAEIIVLKEPPTIRPNSPYDLCSRFAA   1063
USP28_Mouse   EVMRNHWCSYLGKDIAENLQLCLGEFLPRLLDPSAEIIVLKEPPTIRPNSPYDLCNRFAA   1037
USP25_Human   EDMRNRWCSYLGQEMEPHLQEKLTDFLPKLLDCSMEIKSFHEPPKLPSYSTHELCERFAR   1042

USP28_Human   VMESIQGVSTVTVK   1077
USP28_Mouse   VMESIQGVSTVTVK   1051
USP25_Human   IMLSLSRTPADGR-   1055
```

F

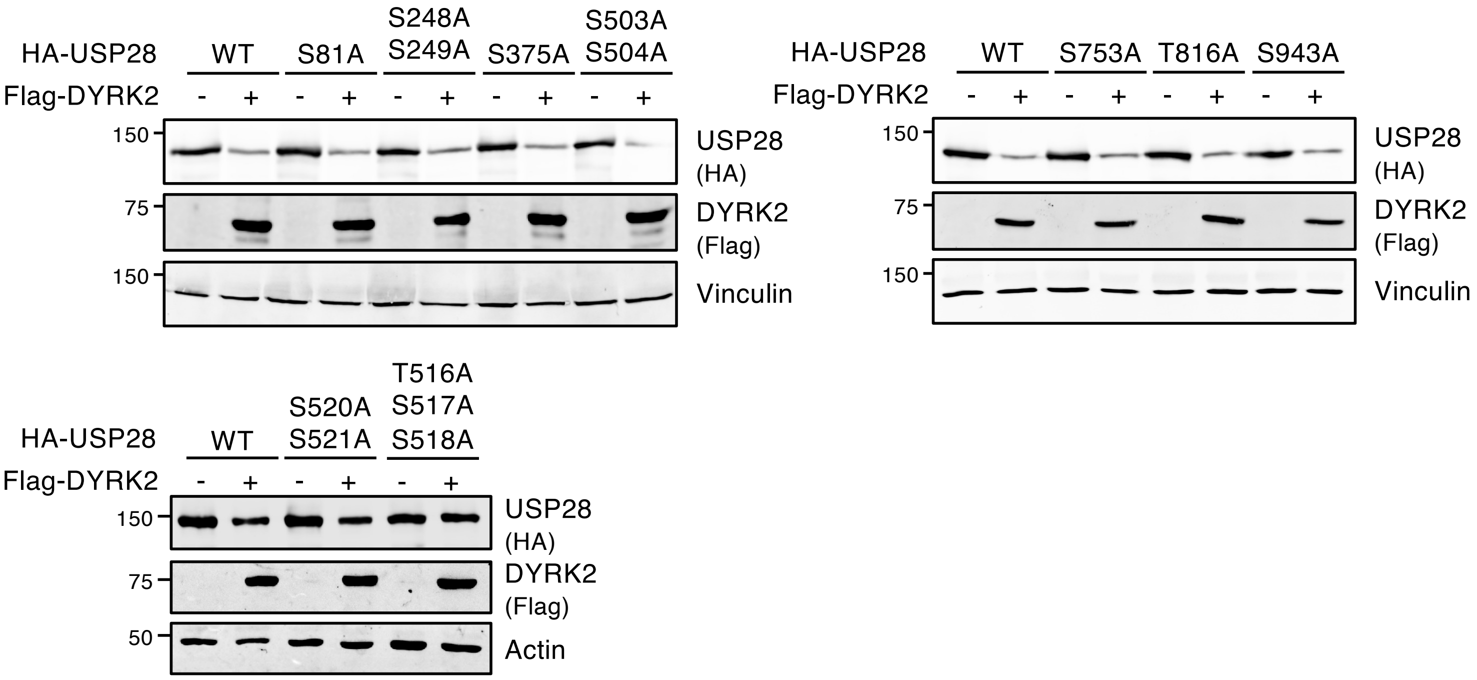

G

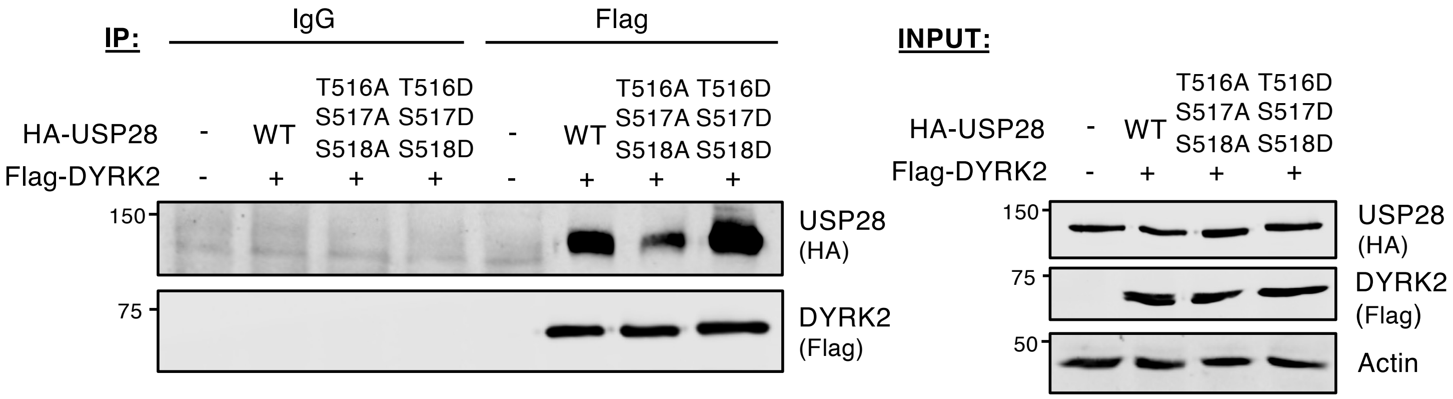

Supp. Figure 2

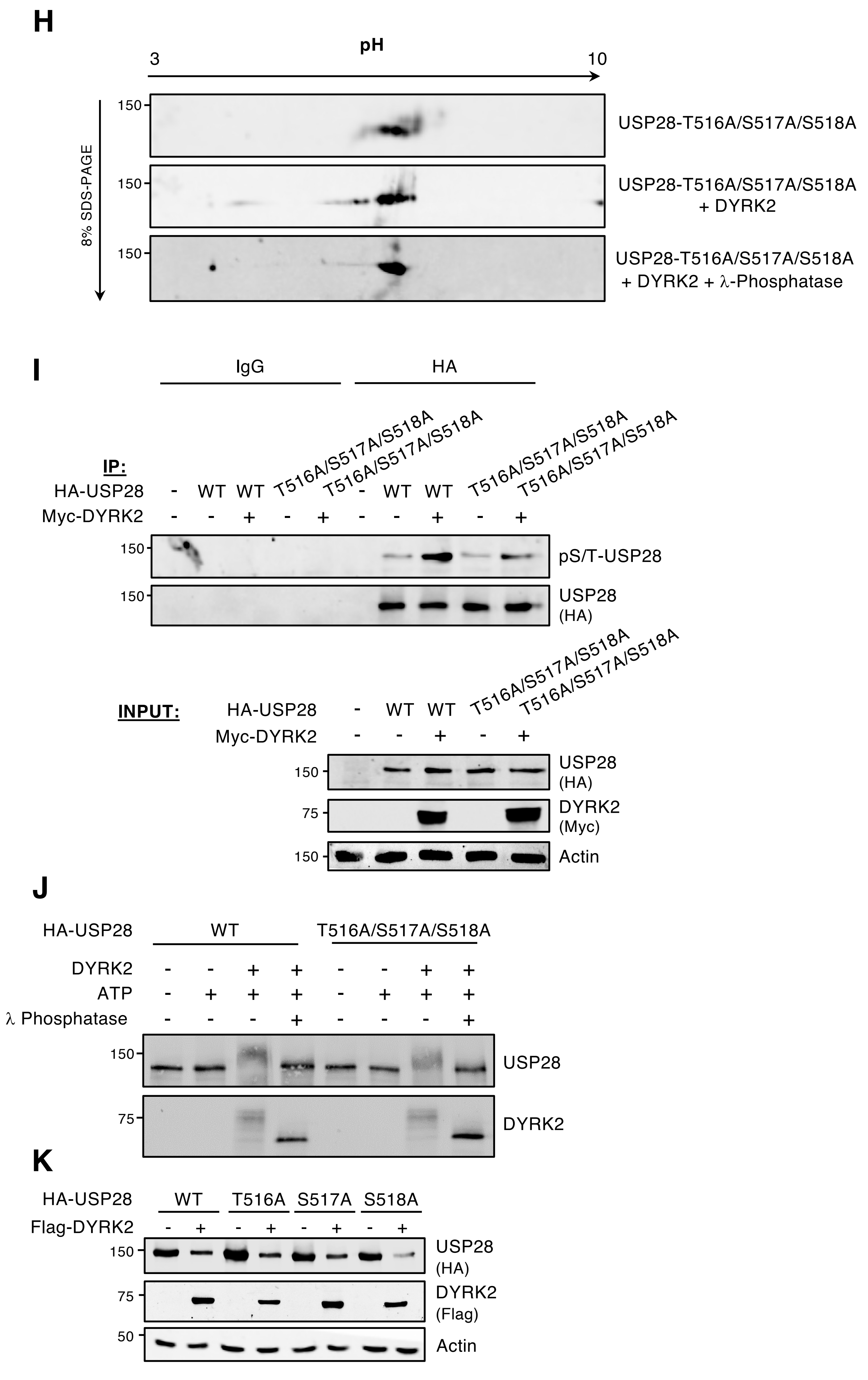

Supp. Figure 3

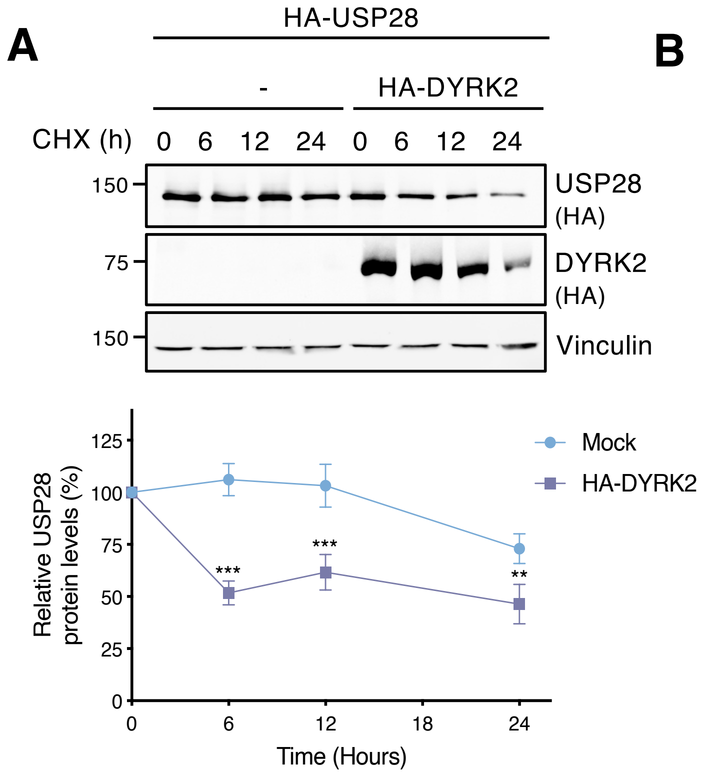

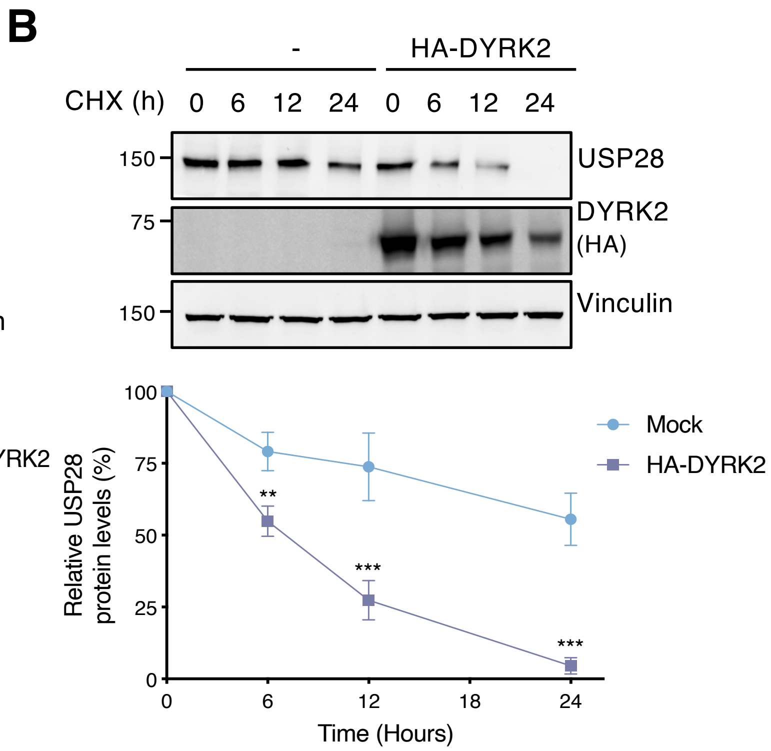

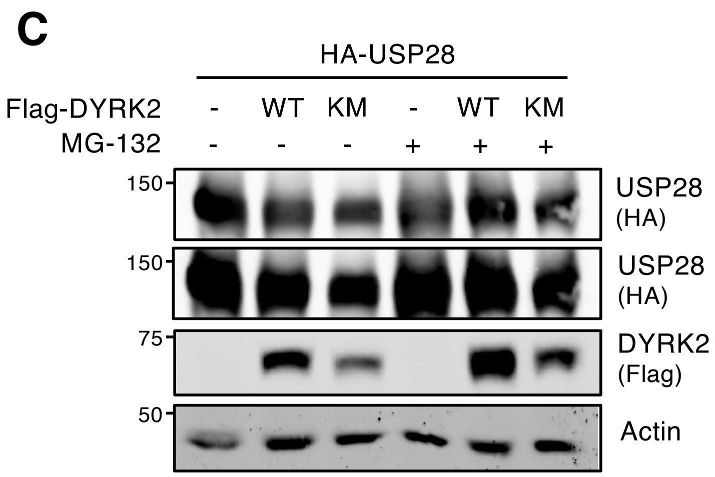

Supp. Figure 4

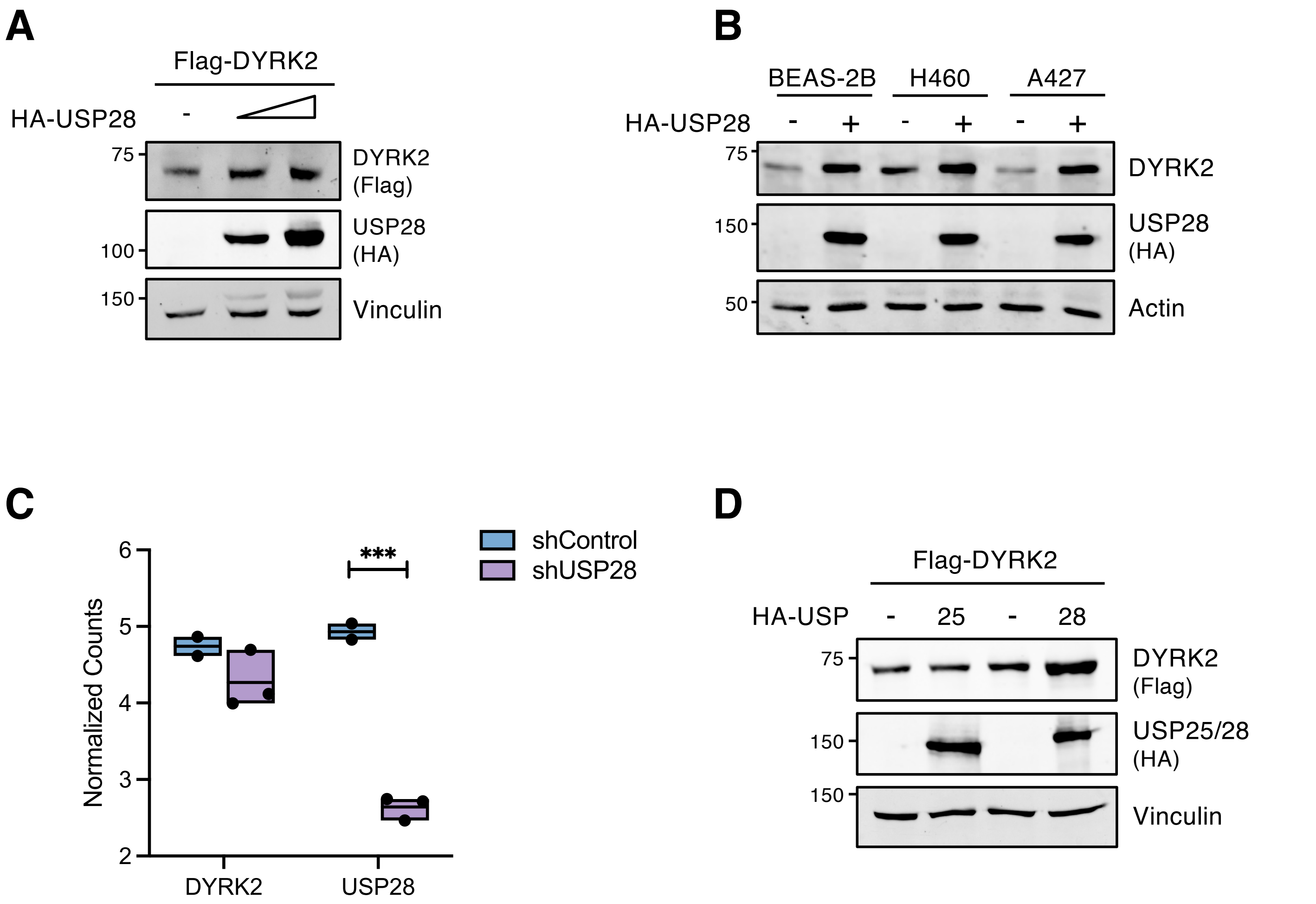

Supp. Figure 5

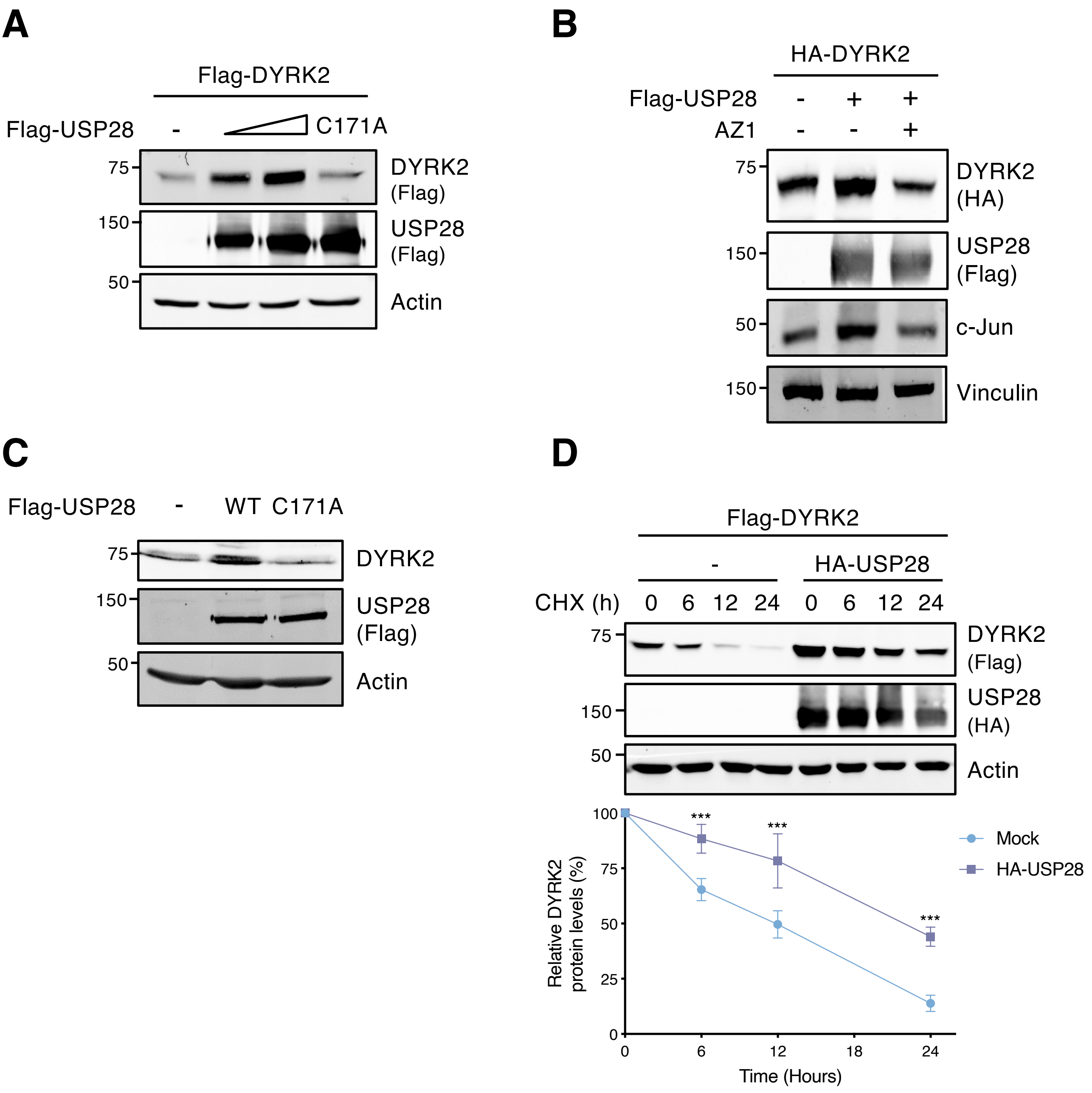

Supp. Figure 6

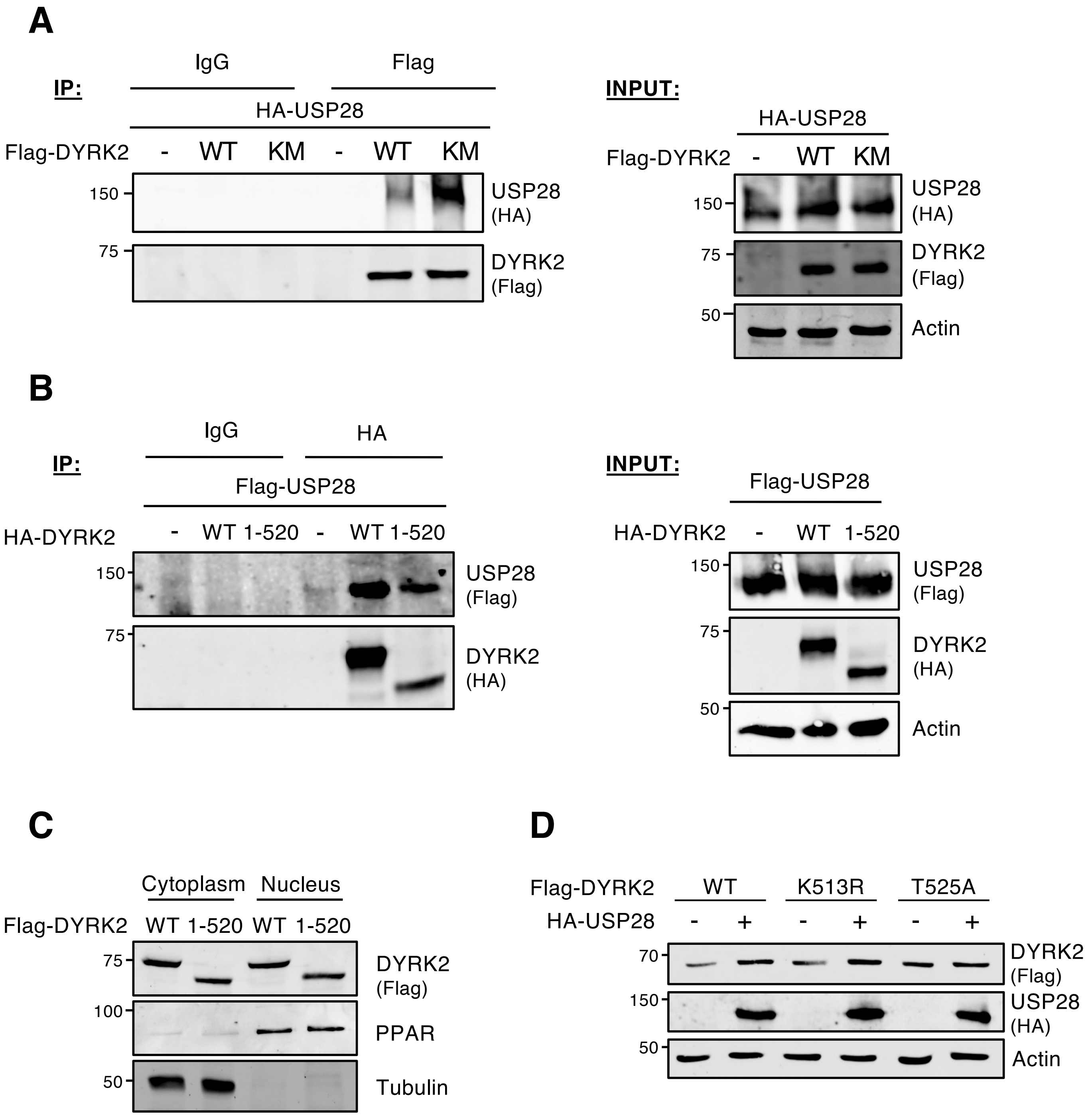

Supp. Figure 7

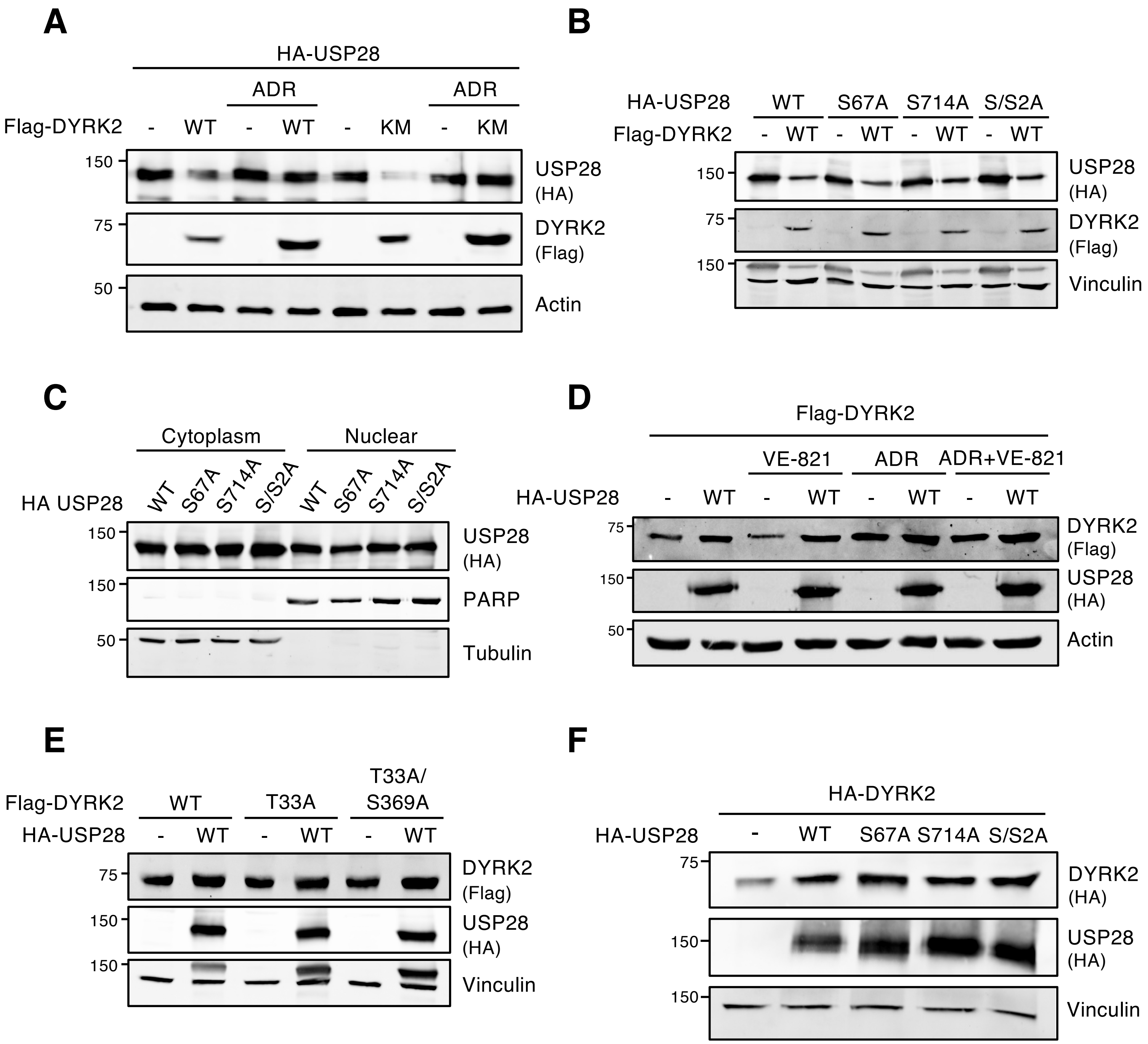

Supp. Figure 8

A

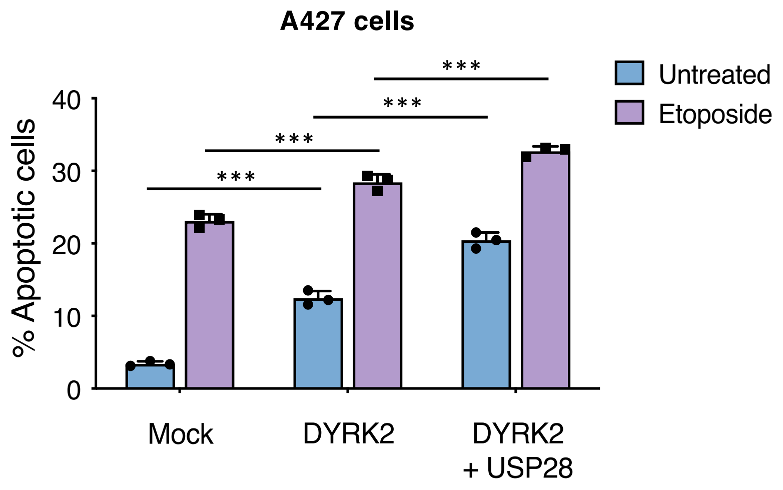

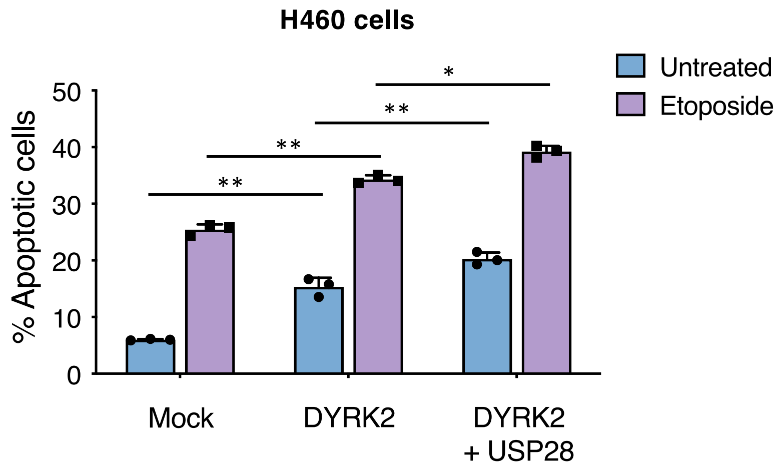

Supplement: Supplementary file 1 — SUPPLEMENTAL MATERIAL [file 41418_2025_1565_MOESM1_ESM.pdf]
