# Supplementary material for: A novel feedback loop between DYRK2 and USP28 regulates cancer homeostasis and DNA damage signaling
Source: Cell Death Differ. 2025 Aug 26;33(1):77–91. doi: 10.1038/s41418-025-01565-w (PMC12811399; doi:10.1038/s41418-025-01565-w)
Supplement: Supplementary file 2 — Supplementary original blots [file 41418_2025_1565_MOESM2_ESM.pdf]

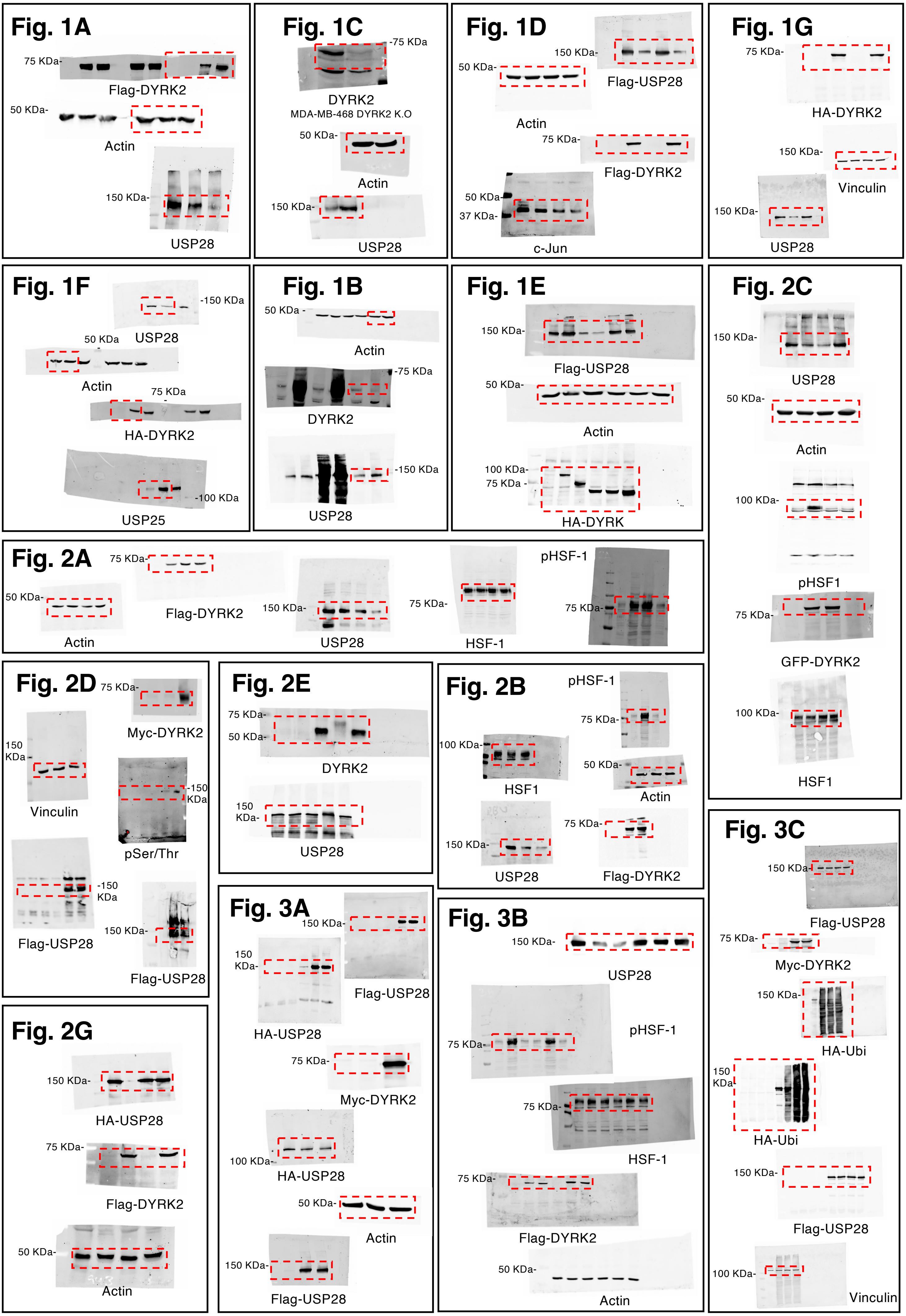

Fig. 1A
75 KDa-
Flag-DYRK2
50 KDa-
Actin
150 KDa-
USP28
Fig. 1C
-75 KDa
DYRK2
MDA-MB-468 DYRK2 K.O
50 KDa-
Actin
150 KDa-
USP28
Fig. 1D
150 KDa-
Flag-USP28
50 KDa-
Actin
75 KDa-
Flag-DYRK2
50 KDa-
37 KDa-
c-Jun
Fig. 1G
75 KDa-
HA-DYRK2
150 KDa-
Vinculin
150 KDa-
USP28
Fig. 1F
-150 KDa
USP28
50 KDa
Actin
75 KDa
HA-DYRK2
-100 KDa
USP25
Fig. 1B
50 KDa -
Actin
-75 KDa
DYRK2
-150 KDa
USP28
Fig. 1E
150 KDa-
Flag-USP28
50 KDa-
Actin
100 KDa-
75 KDa -
HA-DYRK
Fig. 2C
150 KDa-
USP28
50 KDa-
Actin
100 KDa-
pHSF1
75 KDa-
GFP-DYRK2
100 KDa-
HSF1
Fig. 2A
75 KDa-
50 KDa-
Flag-DYRK2
Actin
150 KDa-
USP28
75 KDa-
HSF-1
pHSF-1
75 KDa-
Fig. 2D
75 KDa-
Myc-DYRK2
150 KDa
Vinculin
-150 KDa
pSer/Thr
-150 KDa
Flag-USP28
150 KDa-
Flag-USP28
Fig. 2E
75 KDa-
50 KDa-
DYRK2
150 KDa-
USP28
Fig. 2B
pHSF-1
75 KDa-
100 KDa-
HSF1
50 KDa-
Actin
150 KDa-
USP28
75 KDa-
Flag-DYRK2
Fig. 3C
150 KDa-
Flag-USP28
75 KDa-
Myc-DYRK2
150 KDa-
HA-Ubi
150 KDa
HA-Ubi
150 KDa-
Flag-USP28
100 KDa-
Vinculin
Fig. 3A
150 KDa-
Flag-USP28
150 KDa-
HA-USP28
75 KDa-
Myc-DYRK2
100 KDa-
HA-USP28
50 KDa-
Actin
150 KDa-
Flag-USP28
Fig. 3B
150 KDa-
USP28
pHSF-1
75 KDa-
75 KDa-
HSF-1
75 KDa-
Flag-DYRK2
50 KDa-
Actin
Fig. 2G
150 KDa-
HA-USP28
75 KDa-
Flag-DYRK2
50 KDa-
Actin

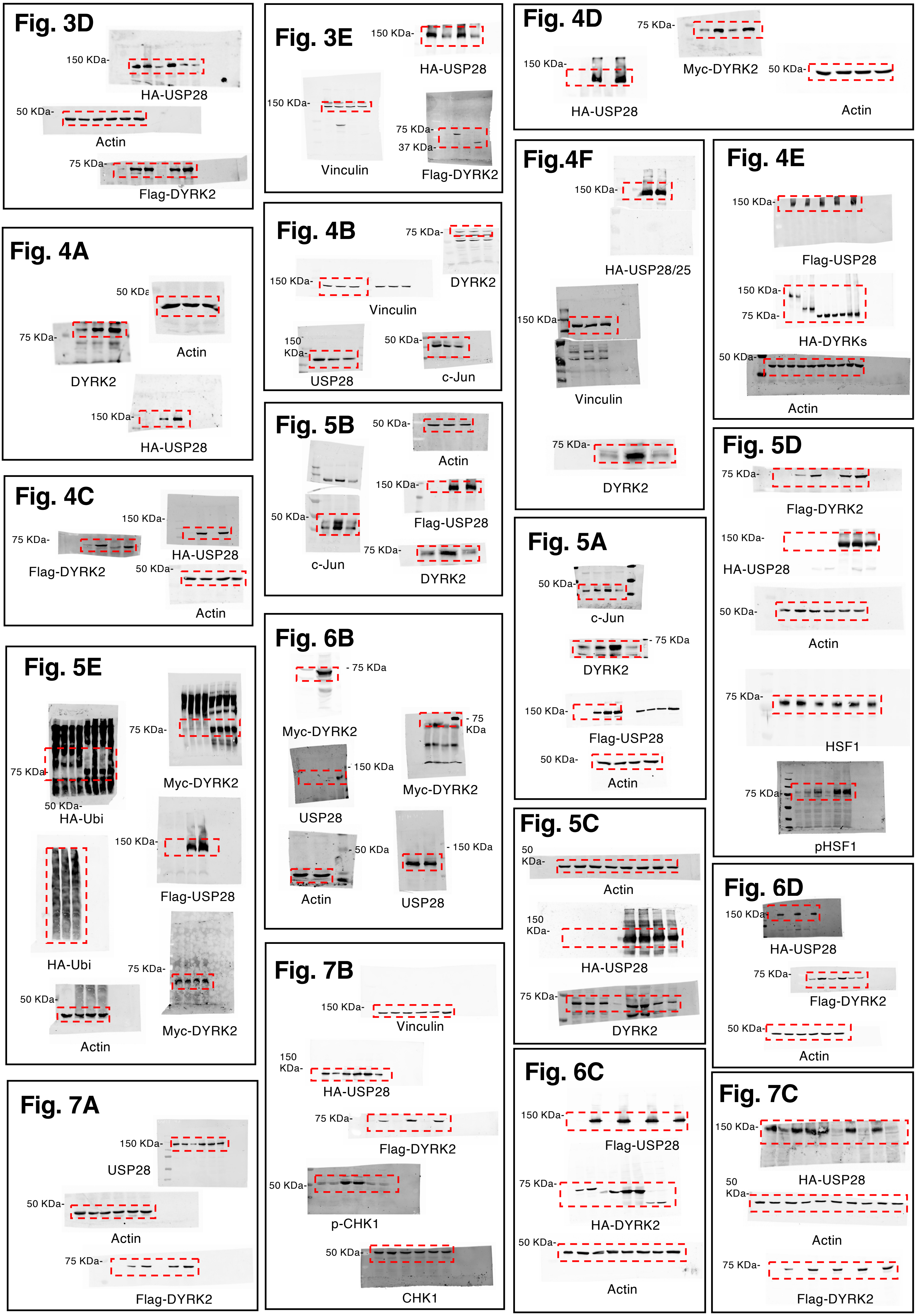

Fig. 3D
150 KDa-
HA-USP28
50 KDa-
Actin
75 KDa-
Flag-DYRK2
Fig. 3E
150 KDa-
HA-USP28
150 KDa-
Vinculin
75 KDa-
37 KDa-
Flag-DYRK2
Fig. 4D
75 KDa-
Myc-DYRK2
150 KDa-
HA-USP28
50 KDa-
Actin
Fig. 4A
50 KDa
Actin
75 KDa-
DYRK2
150 KDa-
HA-USP28
Fig. 4B
75 KDa-
DYRK2
150 KDa-
Vinculin
150 KDa-
USP28
50 KDa-
c-Jun
Fig.4F
150 KDa-
HA-USP28/25
150 KDa-
Vinculin
75 KDa-
DYRK2
Fig. 4E
150 KDa-
Flag-USP28
150 KDa-
75 KDa-
HA-DYRKs
50 KDa-
Actin
Fig. 4C
150 KDa-
HA-USP28
75 KDa-
Flag-DYRK2
50 KDa-
Actin
Fig. 5B
50 KDa-
Actin
150 KDa-
Flag-USP28
50 KDa-
c-Jun
75 KDa-
DYRK2
Fig. 5A
50 KDa-
c-Jun
- 75 KDa
DYRK2
150 KDa-
Flag-USP28
50 KDa-
Actin
Fig. 5D
75 KDa-
Flag-DYRK2
150 KDa-
HA-USP28
50 KDa-
Actin
75 KDa-
HSF1
75 KDa-
pHSF1
Fig. 5E
75 KDa-
75 KDa-
Myc-DYRK2
50 KDa-
HA-Ubi
150 KDa-
Flag-USP28
HA-Ubi
75 KDa-
50 KDa-
Actin
Myc-DYRK2
Fig. 6B
- 75 KDa
Myc-DYRK2
- 150 KDa
USP28
- 75 KDa
Myc-DYRK2
- 50 KDa
Actin
- 150 KDa
USP28
Fig. 5C
50 KDa-
Actin
150 KDa-
HA-USP28
75 KDa-
DYRK2
Fig. 6D
150 KDa-
HA-USP28
75 KDa-
Flag-DYRK2
50 KDa-
Actin
Fig. 7A
150 KDa-
USP28
50 KDa-
Actin
75 KDa-
Flag-DYRK2
Fig. 7B
150 KDa-
Vinculin
150 KDa-
HA-USP28
75 KDa-
Flag-DYRK2
50 KDa-
p-CHK1
50 KDa-
CHK1
Fig. 6C
150 KDa-
Flag-USP28
75 KDa-
HA-DYRK2
50 KDa-
Actin
Fig. 7C
150 KDa-
HA-USP28
50 KDa-
Actin
75 KDa-
Flag-DYRK2

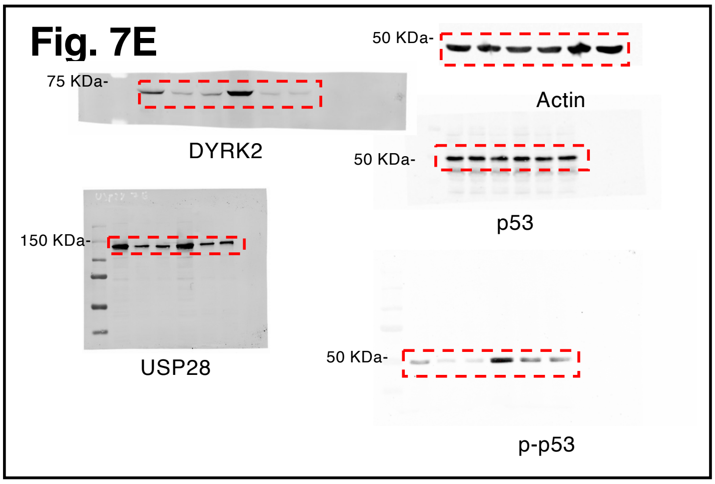
Fig. 7E
50 KDa-
Actin
75 KDa-
DYRK2
50 KDa-
p53
150 KDa-
USP28
50 KDa-
p-p53

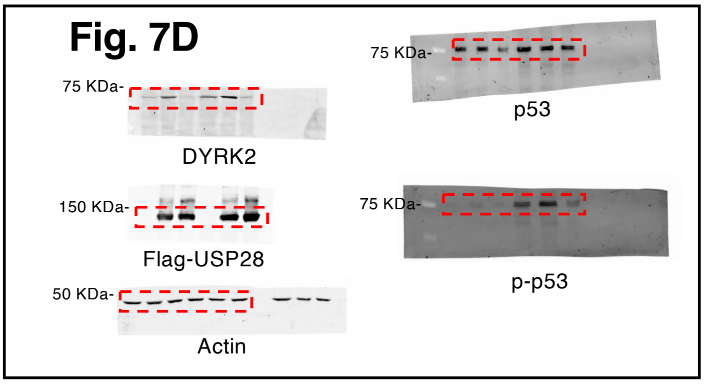
Fig. 7D
75 KDa-
p53
75 KDa-
DYRK2
150 KDa-
Flag-USP28
75 KDa-
p-p53
50 KDa-
Actin

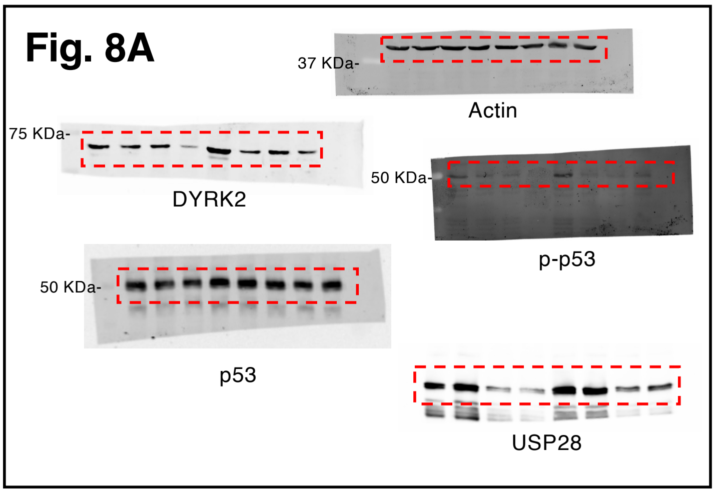
Fig. 8A
37 KDa-
Actin
75 KDa-
DYRK2
50 KDa-
p-p53
50 KDa-
p53
USP28

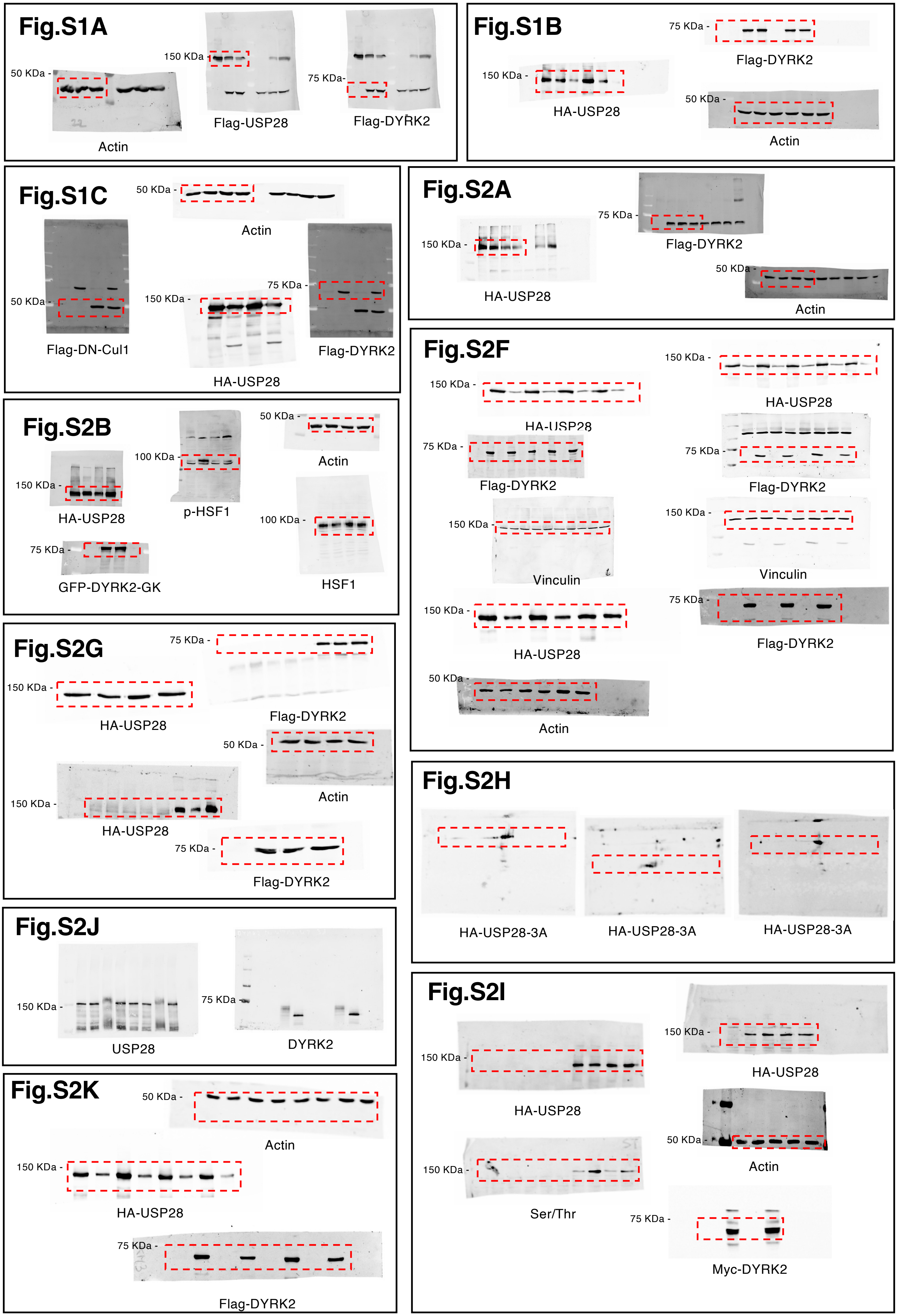
Fig.S1A
50 KDa -
Actin
150 KDa -
Flag-USP28
75 KDa-
Flag-DYRK2
Fig.S1B
75 KDa -
Flag-DYRK2
150 KDa -
HA-USP28
50 KDa -
Actin
Fig.S1C
50 KDa -
Actin
50 KDa -
Flag-DN-Cul1
150 KDa -
75 KDa -
HA-USP28
Flag-DYRK2
Fig.S2A
75 KDa -
Flag-DYRK2
150 KDa -
HA-USP28
50 KDa -
Actin
Fig.S2B
50 KDa -
Actin
100 KDa -
p-HSF1
150 KDa -
HA-USP28
100 KDa -
HSF1
75 KDa -
GFP-DYRK2-GK
Fig.S2F
150 KDa -
HA-USP28
150 KDa -
HA-USP28
75 KDa -
Flag-DYRK2
75 KDa -
Flag-DYRK2
150 KDa -
Vinculin
150 KDa -
Vinculin
150 KDa -
HA-USP28
75 KDa -
Flag-DYRK2
50 KDa -
Actin
Fig.S2G
75 KDa -
150 KDa -
HA-USP28
Flag-DYRK2
50 KDa -
Actin
150 KDa -
HA-USP28
75 KDa -
Flag-DYRK2
Fig.S2H
HA-USP28-3A
HA-USP28-3A
HA-USP28-3A
Fig.S2J
150 KDa -
USP28
75 KDa -
DYRK2
Fig.S2I
150 KDa -
HA-USP28
150 KDa -
HA-USP28
50 KDa -
Actin
150 KDa -
Ser/Thr
75 KDa -
Myc-DYRK2
Fig.S2K
50 KDa -
Actin
150 KDa -
HA-USP28
75 KDa -
Flag-DYRK2

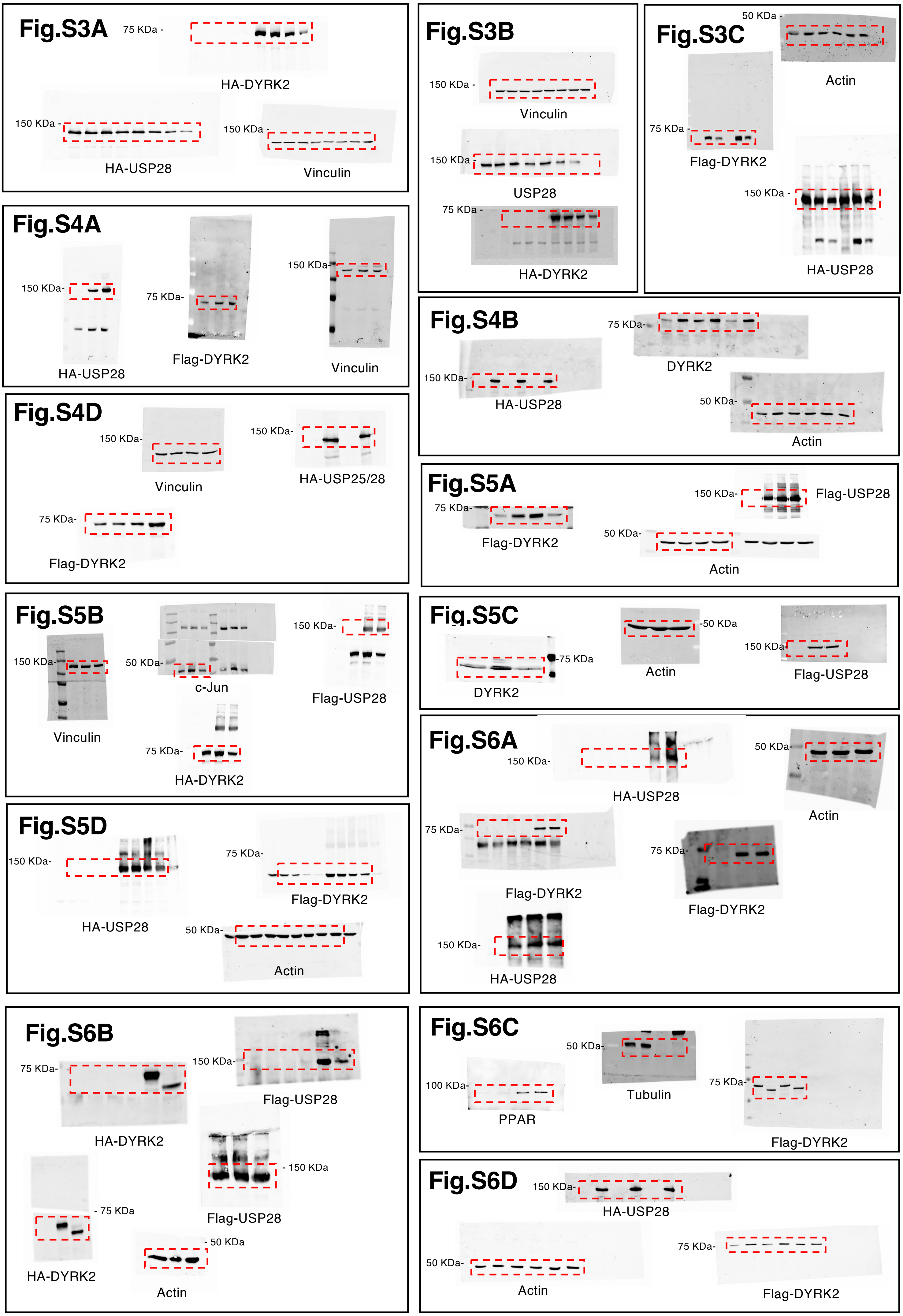
Fig.S3A
75 KDa -
HA-DYRK2
150 KDa -
HA-USP28
150 KDa -
Vinculin
Fig.S3B
150 KDa -
Vinculin
150 KDa -
USP28
75 KDa -
HA-DYRK2
Fig.S3C
50 KDa -
Actin
75 KDa -
Flag-DYRK2
150 KDa -
HA-USP28
Fig.S4A
150 KDa-
HA-USP28
75 KDa-
Flag-DYRK2
150 KDa-
Vinculin
Fig.S4B
75 KDa-
DYRK2
150 KDa-
HA-USP28
50 KDa-
Actin
Fig.S4D
150 KDa-
Vinculin
150 KDa-
HA-USP25/28
75 KDa-
Flag-DYRK2
Fig.S5A
75 KDa-
Flag-DYRK2
150 KDa-
Flag-USP28
50 KDa-
Actin
Fig.S5B
150 KDa-
Vinculin
50 KDa-
c-Jun
150 KDa-
Flag-USP28
75 KDa-
HA-DYRK2
Fig.S5C
-75 KDa
DYRK2
-50 KDa
Actin
150 KDa
Flag-USP28
Fig.S5D
150 KDa-
HA-USP28
75 KDa-
Flag-DYRK2
50 KDa-
Actin
Fig.S6A
150 KDa-
HA-USP28
50 KDa-
Actin
75 KDa-
Flag-DYRK2
75 KDa-
Flag-DYRK2
150 KDa-
HA-USP28
Fig.S6B
75 KDa-
HA-DYRK2
150 KDa-
Flag-USP28
- 150 KDa
Flag-USP28
- 75 KDa
HA-DYRK2
- 50 KDa
Actin
Fig.S6C
100 KDa-
PPAR
50 KDa-
Tubulin
75 KDa-
Flag-DYRK2
Fig.S6D
150 KDa-
HA-USP28
50 KDa-
Actin
75 KDa-
Flag-DYRK2

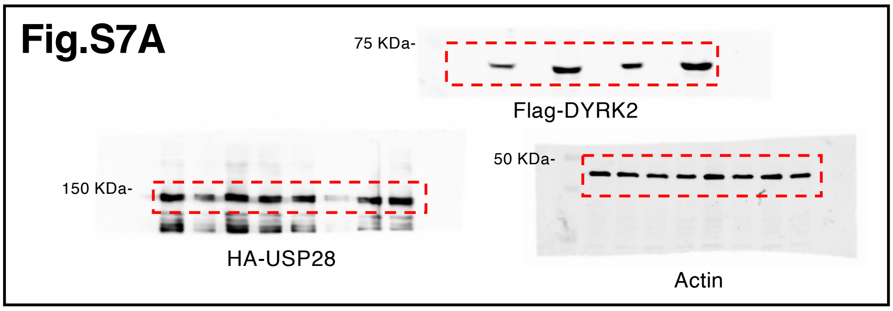
Fig.S7A
75 KDa-
Flag-DYRK2
150 KDa-
HA-USP28
50 KDa-
Actin

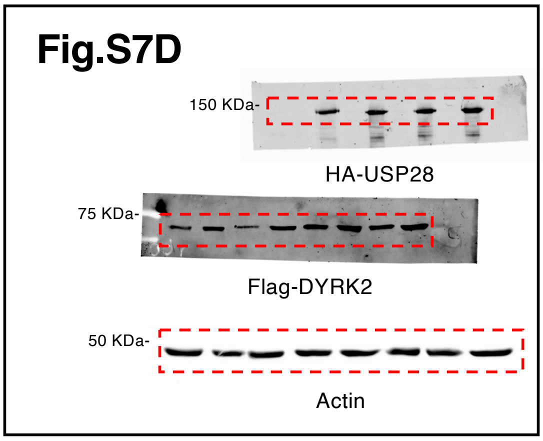
Fig.S7D
150 KDa-
HA-USP28
75 KDa-
Flag-DYRK2
50 KDa-
Actin

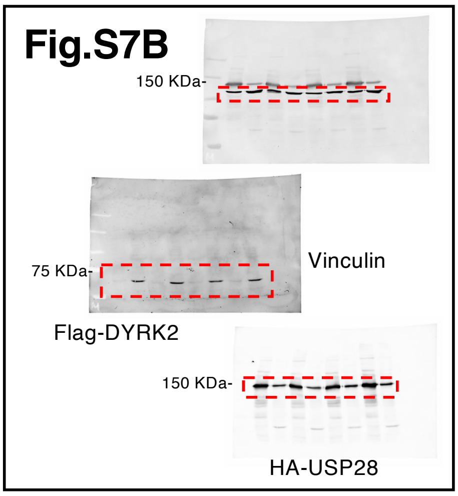
Fig.S7B
150 KDa-
Vinculin
75 KDa-
Flag-DYRK2
150 KDa-
HA-USP28

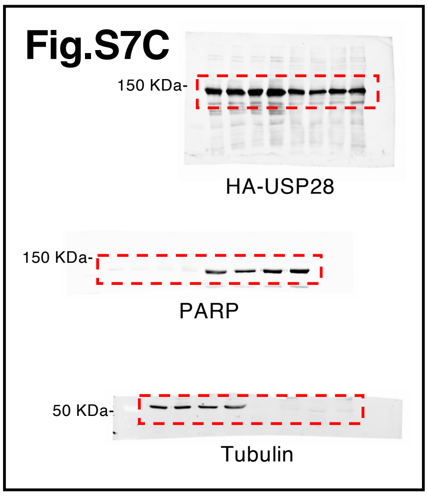
Fig.S7C
150 KDa-
HA-USP28
150 KDa-
PARP
50 KDa-
Tubulin

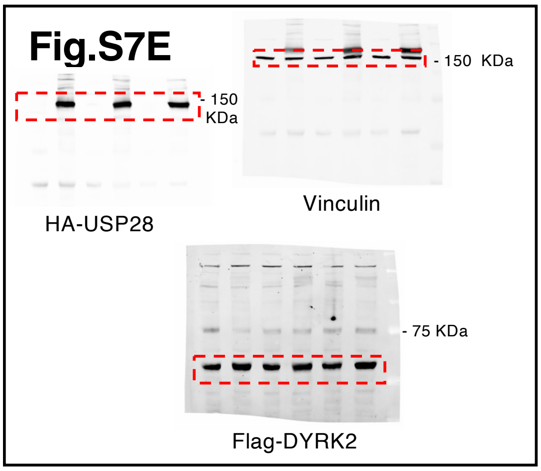
Fig.S7E
- 150 KDa
- 150 KDa
HA-USP28
Vinculin
- 75 KDa
Flag-DYRK2

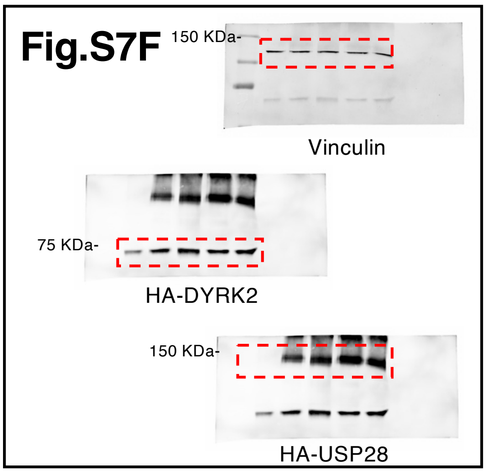
Fig.S7F
150 KDa-
Vinculin
75 KDa-
HA-DYRK2
150 KDa-
HA-USP28
